# Phase Segregation in Cobalt Iron Oxide Nanowires toward Enhanced Oxygen Evolution Reaction Activity

**DOI:** 10.1021/jacsau.1c00561

**Published:** 2022-02-25

**Authors:** Eko Budiyanto, Soma Salamon, Yue Wang, Heiko Wende, Harun Tüysüz

**Affiliations:** † Max-Planck-Institut für Kohlenforschung, Kaiser-Wilhelm-Platz 1, 45470 Mülheim an der Ruhr, Germany; ‡ Faculty of Physics and Center for Nanointegration Duisburg-Essen (CENIDE), University of Duisburg-Essen, 47057 Duisburg, Germany

**Keywords:** cobalt iron oxide, hard templating, electrocatalyst, oxygen evolution reaction, phase transformation, Mössbauer, *in situ* Raman

## Abstract

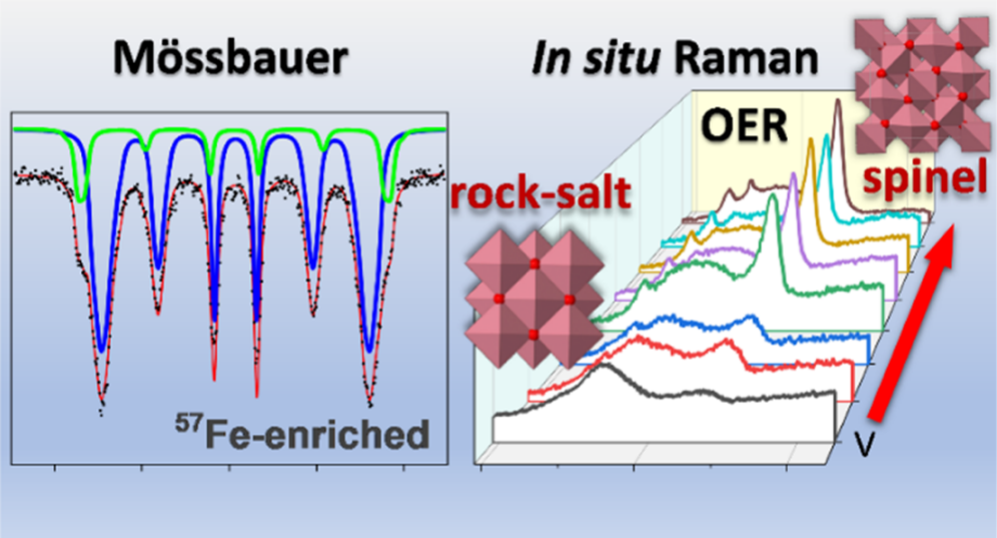

The impact of reduction post-treatment and phase segregation of cobalt iron oxide nanowires on their electrochemical oxygen evolution reaction (OER) activity is investigated. A series of cobalt iron oxide spinel nanowires are prepared via the nanocasting route using ordered mesoporous silica as a hard template. The replicated oxides are selectively reduced through a mild reduction that results in phase transformation as well as the formation of grain boundaries. The detailed structural analyses, including the ^57^Fe isotope-enriched Mössbauer study, validated the formation of iron oxide clusters supported by ordered mesoporous CoO nanowires after the reduction process. This affects the OER activity significantly, whereby the overpotential at 10 mA/cm^2^ decreases from 378 to 339 mV and the current density at 1.7 V vs RHE increases by twofold from 150 to 315 mA/cm^2^. *In situ* Raman microscopy revealed that the surfaces of reduced CoO were oxidized to cobalt with a higher oxidation state upon solvation in the KOH electrolyte. The implementation of external potential bias led to the formation of an oxyhydroxide intermediate and a disordered-spinel phase. The interactions of iron clusters with cobalt oxide at the phase boundaries were found to be beneficial to enhance the charge transfer of the cobalt oxide and boost the overall OER activity by reaching a Faradaic efficiency of up to 96%. All in all, the post-reduction and phase segregation of cobalt iron oxide play an important role as a precatalyst for the OER.

## Introduction

Water electrolysis plays an important role in green hydrogen production and future clean energy technology. The major challenge in this process lies in the oxygen evolution reaction (OER) that occurs at the anode side of the cell. This half-reaction suffers from slow kinetics due to the high energy barrier for four-electron transfer and requires higher overpotential compared to the thermodynamic value of 1.23 V. Therefore, extensive research has been performed within the past decade to develop more effective and robust catalysts for the OER.^1−5^

Owing to high abundancy in nature and competitive price, transition metal-based oxides have been considered as a prime candidate as OER electrocatalysts in alkaline conditions. Among the transition metal-based oxides, cobalt oxide spinel is widely investigated as an OER electrocatalyst due to its stability in harsh alkaline conditions, tunable morphology, crystal, and electronic structures.^6−8^ However, the overall performance of cobalt oxide spinel as an anode material for an alkaline water electrolyzer is still limited due to its low conductivity.^9^ Numerous approaches have been carried out to tune intrinsic properties and enhance the OER activity of cobalt-based oxides. Substitution of di- and trivalent cobalt cations with other heteroelements could tune the electronic structure and conductivity of cobalt oxide that can enhance its OER performance.^10−15^ Coupling of cobalt oxide with more conductive materials and substrates like silver, gold, and carbon has been demonstrated to increase its OER performance as well.^10,16,17^ Recently, we reported that a small amount of iron incorporation into the Co_3_O_4_ lattice structure could boost the OER activity by altering the electronic structure of cobalt on octahedral sites. As a result, the electrical conductivity of the material could be increased, which resulted in a faster charge transfer and higher OER performance.^7,18^

The post-treatments are widely used to tune the OER activity of the electrocatalyst materials by inducing disordering on the nanoscale, such as increased surface area, defect density, and formation of highly active crystalline/amorphous phases. For instance, Jiao et al. reported the formation of a hierarchical structure of mesoporous cobalt oxide using Mg as the sacrificial agent. Through the selective leaching of Mg, the hierarchical mesostructure with a surface area up to 250 m^2^/g could be achieved.^19^ Similarly, our group developed a selective acid leaching protocol to create a cavity in mesostructured cobalt oxide.^20^ As a result, mesostructured cobalt oxide with a fourfold higher surface area could be synthesized. In another method, Waag et al. demonstrated the OER catalytic enhancement of commercial CoFe_2_O_4_ utilizing pulsed laser fragmentation in liquid (PLFL).^21^ Thermal decomposition during the laser irradiation could massively reduce particle size and induce phase transformations toward crystalline CoO and amorphous CoFe_2_O_4_. This structural disorder was proven to improve the charge transport kinetics of the PLFL products compared to the CoFe_2_O_4_ nanoscale educt. Our group applied the PLFL on mesostructured cobalt oxides in a similar direction.^22^ The PLFL could lead to particle fragmentation that significantly increases the surface area and the formation of structural defects (Co^2+^ tetrahedral defect and oxygen vacancies). This laser post-treatment yielded the fragmented cobalt oxide that has superior OER activity compared to the starting material and ordered mesoporous Co_3_O_4_. However, the applied laser also induces the crystal phase transformation into Co_3_O_4_ and CoO crystalline phases.

In another manner, phase boundaries could be introduced into materials via post-treatments. The phase and grain boundaries can provide defects as well as new functionalities for the heterogeneously driven catalytic reactions. Chen et al. reported the synthesis of Co_3_O_4_@CoO nanocubes by reducing the spinel surface with NaBH_4_.^23^ These phase boundaries between CoO and Co_3_O_4_ could lead to the formation of active species that facilitates the chemical reactions without breaking the bulk structure of the electrocatalysts. Suryanto et al. investigated the strong electronic coupling effect between iron oxide and nickel at the interface to enhance the overall water splitting activity.^24^

Even though both post-treatment methods were proven to enhance the catalytic activity of the starting materials, the formation of a spinel and rock-salt biphase is unavoidable due to high-temperature local heating by laser irradiation. Likewise, sacrificial metals (Mg or Mo) could not be totally removed by selective leaching methods. The formation of either biphase cobalt oxides in laser treatment or other metal residues in selective leaching added complexity to the interpretation of catalytic activity enhancement and spectroscopic studies. Hence, a model catalyst with a clean surface is needed to tackle this issue. Regarding the cobalt iron oxide catalyst, the direct assignment of the role of iron in the OER catalytic enhancement was heavily limited by the small proportion of iron compared to cobalt atoms. Hence, the local electronic structure of iron within the spinel structure cannot be directly assessed by XPS or Mössbauer spectroscopy due to the limited sensitivity of the instrument to quantify small amounts of iron.

Herein, we design the small iron oxide clusters supported by ordered mesoporous CoO nanowires. The role of the phase boundaries between iron oxide and cobalt oxide to form an intermediate active state of Co(Fe)-OOH under the OER conditions is investigated by the electrochemical *in situ* Raman microscopy. The mixed oxides are also prepared with ^57^Fe-isotope enrichment to probe the local environment and electronic structure of the iron oxide clusters with Mössbauer spectroscopy without modifying the chemical properties. The reduced samples showed twofold OER catalytic enhancement compared to the spinel educts. This reduced sample was reoxidized to a disordered cobalt oxide spinel phase in the OER resting state and was found to be responsible for the activity enhancement.

## Experimental Section

### Material Synthesis

Cobalt and cobalt iron oxides spinel nanowires were synthesized by the nanocasting method with SBA-15 aged at 100 °C as a hard template.^7,25^ The precursor solutions were prepared by dissolving stoichiometric amounts of Co(NO_3_)_2_·6H_2_O and/or Fe(NO_3_)_3_·9H_2_O (Sigma-Aldrich, ACS reagent, 98% purity) in ethanol. After the first impregnation of metal precursors into the SBA-15 template, the composite was dried slowly at 40 °C overnight and calcined at 250 °C for 4 h in air. The final calcination was performed after the second impregnation and carried out at 500 °C for 4 h in an air atmosphere. The ramping rate for calcination was 2 °C/min. The SBA-15 template was then etched with hot 2 M NaOH solution. The initial spinel materials (Co_3–*x*_Fe*_x_*O_4_) were further labeled as Co_3_O_4_, Co/Fe 32, and Co/Fe 3.

FeO_*x*_-CoO samples were synthesized from prepared cobalt iron oxide nanowires via post-treatment using a mild reduction process.^26−28^ Then, 200 mg of sample were placed inside a tube furnace and kept in an ethanol/N_2_ atmosphere. The ethanol/N_2_ flow was generated by purging N_2_ gas (100 mL/min) into liquid ethanol in a round-bottom flask. The thermal treatment was performed at 270 °C for 4 h (ramping rate of 2 °C/min). The samples after reduction (FeO*_x_*–CoO) were further denoted as CoO, Co/Fe 32-red, and Co/Fe 3-red. Mesoporous Fe_3_O_4_ (magnetite) was also prepared as a control sample by applying similar reduction post-treatment on mesoporous α-Fe_2_O_3_ (hematite) as a starting material. The control experiment with hydrogen reduction was performed in a tubular furnace with 5% H_2_ flow in Ar (100 mL/min) at 270 °C for 2 h (ramping rate 2 °C/min).

^57^Fe isotope-labeled cobalt iron oxides were prepared using a similar procedure. Instead of Fe(NO_3_)_3_·9H_2_O, the iron precursor was prepared by diluting ^57^Fe-isotope powder (Chemotrade GmbH, 95% enrichment) in a stoichiometric amount of HNO_3_:distilled water (1:1) solution. These samples after reduction in ethanol/N_2_ were further denoted as Co/^57^Fe 32-red and Co/^57^Fe 3-red.

### Material Characterization

Powder X-ray diffraction (XRD) data of the as-prepared samples were recorded with an STOE theta/theta diffractometer operating in a reflection mode with a Cu Kα_1,2_ radiation X-ray source (λ: 1.5406 Å) using a step size of 0.02° 2θ. The instrument was equipped with an energy discriminating detector. For the sample deposited on carbon fiber paper, the X-ray powder patterns for qualitative phase analysis were collected on a Stoe STADI P transmission diffractometer using Mo radiation (0.7093 Å). The instrument is equipped with a primary Ge (111) monochromator (Mo Kα_1_) and a position-sensitive Mythen1K detector. The samples were mounted on a transmission sample holder. Data were recorded in the range of 2–40° 2θ with a step width of 0.015° 2θ and 60 s per step of data acquisition. For each sample, 8 scans were collected and summed after data collection. Phase identification was referred to the experimental data in the PDF-2 ICCD database.^29^ Fourier-transform infrared (FTIR) spectra were recorded with a Perkin-Elmer Spectrum Two spectrometer with 32 scan spectra acquisition.

The Brunauer–Emmett–Teller (BET) surface area was calculated in the relative pressure range (*p*/*p*°) of 0.06 to 0.3 with a nitrogen physisorption measurement that was carried out using a 3Flex Micromeritics instrument at 77 K. Pore size distribution was calculated with the Barrett, Joyner, and Halenda (BJH) method from the desorption branch of the isotherms. Prior to the measurement, the powder samples were degassed under vacuum at 120 °C for 10 h.

High-resolution transmission electron microscopy (HR-TEM) images were recorded with a cold field-emission gun (FEG) Hitachi HF2000 operating at a 200 keV acceleration voltage. Secondary electron (SE) and dark field images were recorded with a Hitachi HD-2700 Cs-corrected dedicated scanning transmission electron microscope (STEM) equipped with a cold field-emission gun operated at 200 keV. The energy-dispersive X-ray spectroscopy (EDX) elemental mapping and line scan analysis were performed with an EDAX Octane T Ultra W 200mm2 SDD. For samples deposited on carbon fiber paper, scanning electron microscopy (SEM)-EDX mapping was recorded with a Hitachi S-3500N equipped with a Si(Li) Pentafet plus detector from Oxford instruments.

The inductively coupled plasma-optical emission spectrometry (ICP-OES) measurement was carried out with a SPECTROGREEN instrument, and the electrolyte solution samples were taken from the electrochemical cell before and after the reaction. X-ray photoelectron spectroscopy (XPS) data were measured with a SPECS spectrometer with a hemispherical analyzer (PHOIBOS 150). The monochromatized Mg Kα X-ray source (*E* = 1254.6 eV) was operated at 50 W/10 kV. The narrow scans were measured with 20 eV pass energy. The lens mode was set to the medium area, and the analysis chamber was 5 × 10^–10^ mbar. The binding energy was corrected for surface charging using the C 1s peak for contaminant carbon as a reference at 284.5 eV.

Mössbauer spectra were recorded in transmission geometry on powder samples, using a constant acceleration Mössbauer driving unit with a ^57^Co source embedded in an Rh matrix. α-Fe foil measured at room temperature was used as a reference sample to calibrate the spectrometer. The samples were kept in a homogenous magnetic field of 5 T, applied parallel to the γ-ray propagation direction with the aid of a liquid helium bath cryostat containing a superconducting magnet in split-pair geometry. Temperature-dependent magnetization measurements were performed between 5 and 400 K at 10 mT following the zero-field-cooled field-cooled (ZFC-FC) protocol, while field-dependent M(H) sweeps were recorded at 5 and 300 K at field amplitudes of up to 9 T. All measurements were completed using the vibrating sample magnetometer (VSM) option of a Quantum Design PPMS DynaCool.

### Electrochemical Measurement

The electrochemical oxygen evolution reaction (OER) was measured with a VSP-300 BioLogic potentiostat and a rotating disc electrode (RDE) system (Model: AFMSRCE, PINE Research Instrumentation). The RDE was operated with a 3 electrode system, using Pt wire as the counter electrode, a reference hydrogen electrode (HydroFlex, Gaskatel) as the reference electrode, glassy carbon as a working electrode, and 200 mL of 1 M KOH solution as the electrolyte. Prior to the measurement, the cell was saturated with Ar flow. The electrochemical cell was kept at 298 K using a thermostat. Working electrodes were prepared by depositing electrocatalyst ink onto glassy carbon (GC) electrodes (5 mm diameter, 0.196 cm^2^ geometric surface area). GC electrodes were polished with Al_2_O_3_ slurry (1 and 0.05 μm, Buehler).

The ink was prepared by dispersing 4.8 mg of electrocatalyst in 0.75 mL of H_2_O, 0.25 mL isopropanol, and 50 μL of Nafion 117 (Sigma-Aldrich). The dispersion was then sonicated for 30 min to obtain a homogeneous ink. Finally, 5.25 μL of ink was drop-casted onto a clean GC electrode surface and dried (calculated catalyst loading was equal to 0.12 mg/cm^2^).

For postmortem analysis, the sample was deposited onto 1 cm^2^ of carbon fiber paper (Toray TGP-H-60 non–PTFE coated). After prolonged immersion in 1 M KOH or chronopotentiometry, the working electrode was dipped in ultrapure water, dried, and protected under an Ar atmosphere.

Linear scan voltammetry (LSV) curves were recorded at a scan rate of 10 mV/s within the 0.7 to 1.7 V vs RHE. Cyclic voltammetry (CV) was measured at a scan rate of 50 mV/s within the 0.7 to 1.6 V vs RHE potential window. The stability test was conducted with controlled current (chronopotentiometry) at 10 mA/cm^2^ of geometric current density. Electrochemical impedance spectroscopy (EIS) was measured within the 100 mHz–100 kHz frequency range at 1.6 V vs RHE and 5 mV of amplitude. Experimental Nyquist plots were then fitted to the equivalent circuit model using the Z-Fit feature in EC-Lab software. The IR drop was compensated at 85%.

Faradaic efficiency (FE) measurement was carried out with a VSP-300 bipotentiostat mode and rotating ring-disc electrode (RRDE) method. A glassy carbon disc and Pt ring were used as the RRDE tip (model: E7R9, Pine Research Instrumentation, glassy carbon disc outer diameter: 5.61 mm). The collection efficiency (*N* = 37.7%) of the RRDE was measured by oxidizing K_4_Fe(CN)_6_ solution at the disc and performing reduction at the Pt ring. The RRDE was cleaned, and the sample was deposited in the same manner for the RDE working electrode preparation. To measure the ring background current, the disc electrode was held at an open-circuit potential for 2 min, while the Pt ring was biased at 0.4 V vs RHE. The rotation rate was kept at 1600 rpm, and the 1 M KOH electrolyte was saturated with Ar flow for 30 min prior to each measurement step.

Raman measurements were carried out with an Invia Renishaw Raman microscope equipped with 532 nm of laser excitation wavelength, 1800 l/mm grading, and coupled with a 50× objective lens (Leica). The *ex situ* Raman spectra were collected at 0.25 mW laser power. The *In situ* Raman measurement was performed in a customized *in situ* electrochemical flow cell. Pt wire and the hydrogen electrode (HydroFlex, Gaskatel) were used as counter and reference electrodes, respectively. The sample ink was then drop-casted on top of the rough Au foil and utilized as the working electrode. The Au foil was roughened following the previously reported protocol.^30,31^ During the measurement, Ar-saturated 1 M KOH flux was controlled with a peristaltic pump with a flow rate of 1–8 mL/min, depending on the bubble generation inside the cell. The sample was held at open-circuit potential (OCP) for 15 min prior to the measurement. Ten consecutive scans with 3 s exposure time at 0.5 mW laser power were performed to measure *in situ* Raman spectra. The Raman spectra were recorded *in situ* in a chronoamperometric (CA) mode with potential held for 1 min at OCP and 0.1 V gradual potential bias from 1.0 to 1.5 V vs RHE.

## Results and Discussion

The ordered mesoporous Co_3_O_4_ and iron-incorporated cobalt oxide nanowires (with atomic ratios of Co/Fe 32 and Co/Fe 3) were prepared via the nanocasting method using SBA-15 as a hard template. These two ratios were chosen to examine the impact of the dilute amount and a large amount of iron substitution (where the cobalt and cobalt iron spinels’ miscible phase is expected) on the OER activity. Their detailed structural analyses are provided in our previous study.^7^ Ordered mesoporous CoO and iron oxide clusters supported by ordered mesoporous CoO nanowires were synthesized by the reduction of Co_3_O_4_ and cobalt iron oxide spinel nanowires using ethanol/N_2_ gas flow as a mild and selective reducing agent. In this reducing atmosphere, Co^3+^ at octahedral sites of spinel was expected to be reduced into Co^2+^, resulting in a rock-salt CoO structure. Subsequently, Fe^3+^ that preferred to occupy octahedral sites in the cobalt iron oxide spinel lattice (as suggested by extended X-ray absorption fine structure/EXAFS spectra from our previous work) was partially reduced into Fe^2+^, creating a stable cluster of iron oxide.^7^

**Figure 1:**
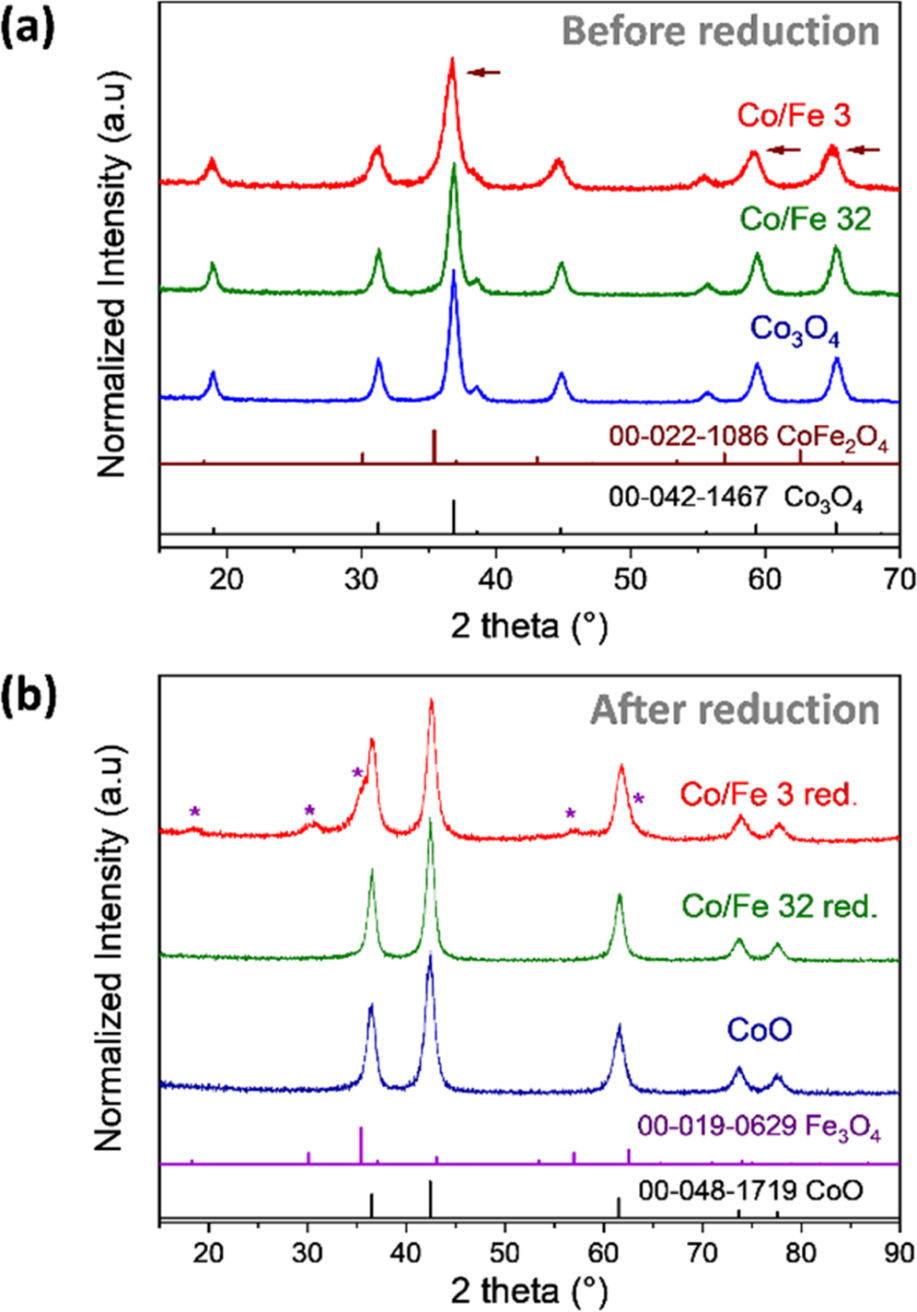
Powder XRD pattern of sample series (a) before reduction and (b) after reduction in an ethanol/N_2_ atmosphere. The XRD data was measured with a Cu Kα_1,2_ radiation X-ray source (λ: 1.5406 Å).

The crystalline phase of the materials before and after reduction was evaluated with wide-angle powder XRD in a reflection mode (Figure 1a,b). A clear phase transformation from a cobalt oxide spinel (PDF 00–042–1467) into the rock-salt structure (PDF 00–048–1719) is observed for all materials. Additionally, a crystalline phase corresponding to magnetite (Fe_3_O_4_, PDF 00–019–0629) evolves after reduction in Co/Fe 3-red. This indicates that the Fe^3+^ in the spinel lattice was partly reduced into Fe^2+^, creating a magnetite cluster. The magnetite phase might also be generated on Co/Fe 32-red; however, the magnetite reflections, in this case, are barely visible due to the low amount of iron (2.3 mass % compared to the total mass of the material).

Nitrogen physisorption is performed to measure the textural parameters of pristine samples and after the reduction. The adsorption–desorption hysteresis loop and pore size distribution of both sample series are depicted in Figure S1a–d. The samples before and after reduction show typical mesoporous materials as depicted by type IV isotherm (Figure S1a,b).^32^ However, the BET surface area decreases slightly from 116–125 to 95–96 m^2^/g after the reduction. The decrease of the surface area after reduction could be attributed to the volume shrinkage of cobalt oxide nanowires due to the mass density difference between Co_3_O_4_ (6.11 g/cm^3^) and CoO (6.44 g/cm^3^).^26^ In addition, the samples have identical pore size distribution before and after reduction (Figure S1c,d), indicating that the pore structures of the nanowire material were retained after heat treatment and reduction at 270 °C.

**Figure 2:**
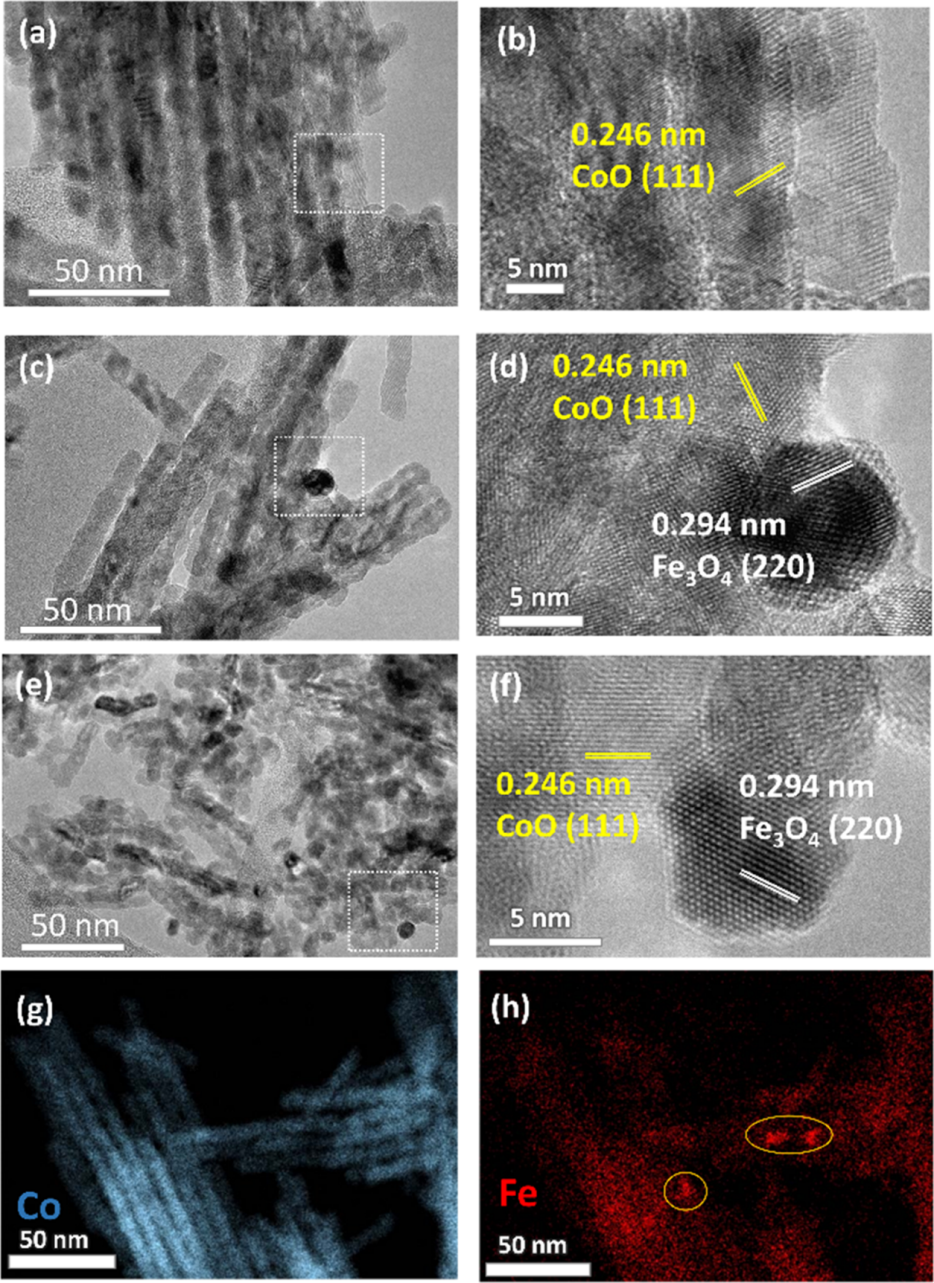
TEM micrographs of samples after reduction (a) CoO, (b) HR-TEM of CoO, (c) Co/Fe 32-red, and (d) HR-TEM of Co/Fe 32-red. (e) Co/Fe 3-red, (f) HR-TEM of Co/Fe 3-red. HR-TEM images were recorded in the region marked by white boxes. Cross-sectional EDX elemental mapping of Co/Fe 32-red on (g) Co and (h) Fe.

Electron microscopy studies were then carried out to visualize the morphology of materials after reduction as well as the formation of iron oxide clusters within the nanowire’s matrix. A highly ordered nanowire arrangement is observed in CoO, and shorter nanowire arrays could be found in the sample that contained iron (Figure 2a–f). The less ordered mesostructured and shorter nanowire arrays by incorporation of iron in the pristine samples (Figure S2a,b) are caused by the different thermal decomposition temperatures between the cobalt and iron nitrate precursor. This triggers the iron oxide moieties to block the interconnected micropores during the calcination and hinders the formation of a perfect negative replica.^7^ After the reduction, the pore structure and nanowire morphology were retained, consistent with the finding from nitrogen physisorption. The high-resolution image (Figure 2d,f) reveals the formation of crystalline phase boundaries between the nanoparticles and nanowires, indicating that the nanoparticles (iron oxide cluster) are supported on the CoO nanowires matrix. With the increasing amount of iron, as expected, the observed iron oxide clusters within the sample increased accordingly (Figure 2c,e).

The surface topology of the samples after reduction was further investigated with scanning transmission electron microscopy (STEM) imaging (Figure S3a–d). Even though the nanowire arrays were maintained, the surface of the nanowires became rough after reduction. This could be attributed to the iron moieties, which left the spinel lattice and created iron oxide clusters. EDX elemental mapping was then performed on the cross-sectioned sample to visualize the iron and cobalt distribution within the nanowire matrix. As depicted in Figure 2g, cobalt was distributed evenly within the nanowire’s structure. On the other hand, localized iron clusters were observed in several areas, as indicated by the yellow circles in Figure 2h.

To probe the local environment and electronic structure of iron moieties, ^57^Fe isotope-enriched samples were prepared to enhance the quality of Mössbauer spectra for the nanomaterials with low iron content. The characterization data of ^57^Fe isotope-labeled samples are presented in Figure S4a–f. The XRD patterns showed a similar phase transformation from a cobalt iron oxide spinel into the rock-salt structure and Fe_3_O_4_ after the mild reduction process. TEM imaging revealed the absence of big aggregates, and EDX analysis showed the actual atomic ratio of cobalt to iron. Altogether, a similar synthesis result could be reproduced via the substitution of the Fe precursor with the ^57^Fe isotope counterpart.

The magnetic properties of the spinel and rock-salt system were then measured to investigate the effect of phase transformation after reduction (Figure S5a–c). A striking difference could be observed in the zero-field-cooled (ZFC) curves of Co/Fe 32 and Co/Fe 3 before and after reduction (Figure S5a,b). The Co/Fe 32 spinel showed a low Néel temperature of 23.5 K that is similar to the Co_3_O_4_ nanoparticles with antiferromagnetic behaviors.^33^ The Néel temperatures increase significantly for the samples after reduction, approaching around 270 K, which is close to the Néel temperature of CoO.^34,35^ Similar behavior could also be observed for Co/Fe 3 before and after reduction. At low temperatures, the discrepancy between rock-salt/wustite-like and Co-rich spinel phases is challenging, as both are expected to be antiferromagnetic. In the 4 K M(H) curves (Figure S5c), we can, however, still see typical behavior of a system going from a Co-rich phase, predominantly antiferromagnetic Co_3_O_4_ or CoO, to a more Fe-rich one, with the resulting increase in magnetization, remanence, and coercivity.

**Figure 3:**
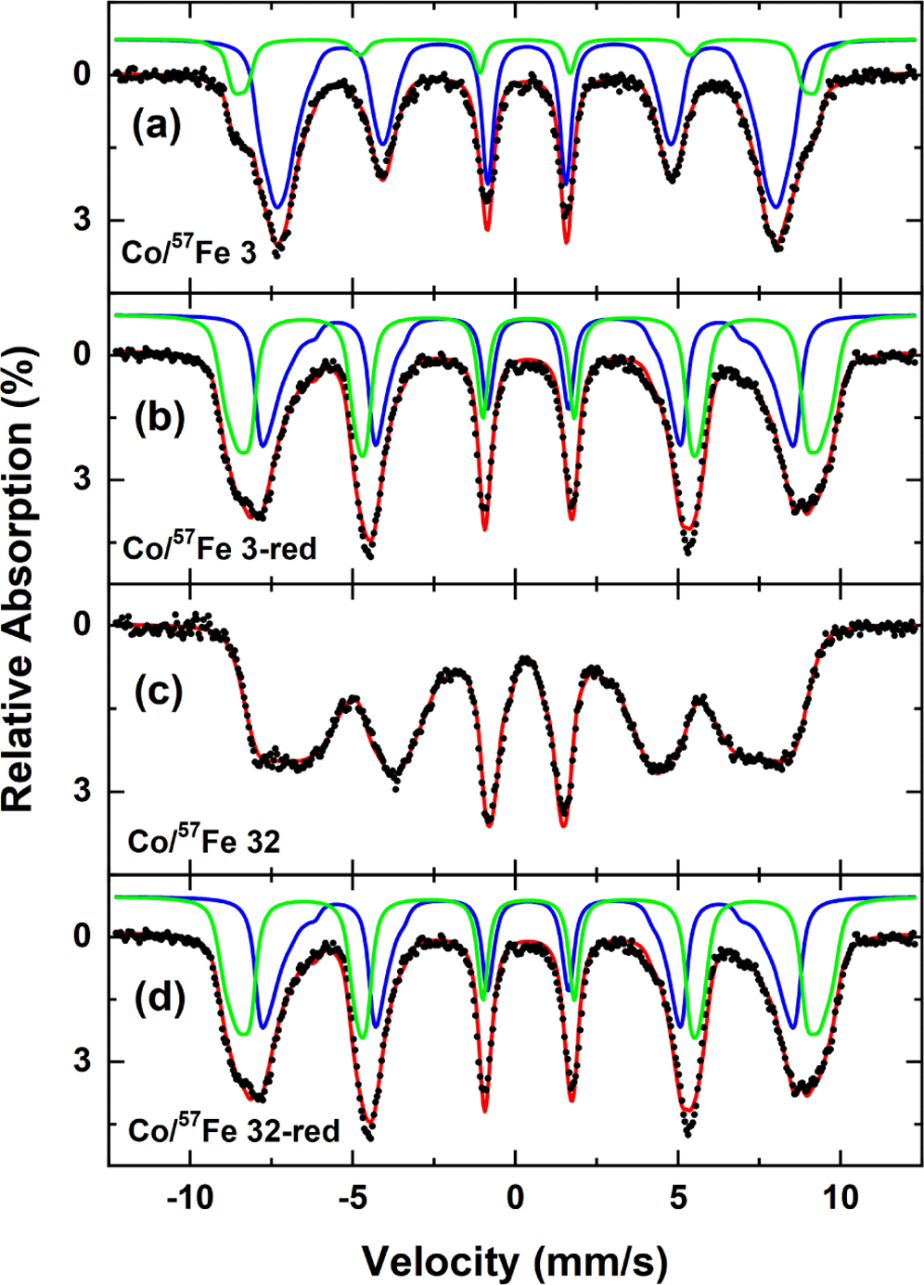
Mössbauer spectra of ^57^Fe isotope-labeled samples: (a) Co/^57^Fe 3, (b) Co/^57^Fe 3-red, (c) Co/^57^Fe 32, and (d) Co/^57^Fe 32-red measured at 5 T and 4.3 K. The subspectra correspond to tetrahedrally coordinated A-sites (green) and octahedral B-sites (blue).

Mössbauer spectra were then measured at a 5 T magnetic field parallel to the γ-ray propagation direction and 4.3 K to determine the inversion parameter as well as the difference in the local iron environment before and after reduction. Here, the relative spectral areas of the respective subspectra can be assumed as proportional to the amount of Fe ions on the tetrahedrally coordinated A-site and the octahedrally coordinated B-site.^36^ The inversion parameter is used to differentiate the normal (0) and a fully inverse spinel (1) phases; however, one has to consider that the low amount of Fe in our samples does not permit the direct determination of the resulting distribution of Co ions displaced by Fe. To avoid any confusion, we will still refer to the calculated number as the inversion parameter, but it should only be regarded as an indicator for the Fe site occupation preferences. As seen in Figure 3a, the tetrahedral A-site is strongly diminished for the Co/^57^Fe 3 spinel, with the great majority of Fe ions having moved over to the B-site. At this point, the inversion parameter was much closer to a normal spinel at ca. 0.35 compared to the inversion degree of 0.65 that was found for the CoFe_2_O_4_ reference sample (Figure S6). This result is consistent with our finding from EXAFS measurement, where the Co^2+^/Co^3+^ ratio increased with the incorporation of iron.^7,37^ Hence, it can be concluded that Fe^3+^ preferred to substitute ions on the octahedral site in the spinel lattice. In addition, it indicates that while the iron ion distribution has strongly shifted, the spin canting angle of ca. 41° (determined from the ratio of lines 2 and 3) has not significantly changed in comparison with the reference samples shown in Figure S6.^38^

For the Co/^57^Fe 3-red sample, a strong overlap of the two subspectra was observed, although the presence of individual subspectra is still evident by the broad lines 1 and 6 with the associated fine structure in their peaks (Figure 3b). This sample was calculated to have a higher inversion parameter (ca. 0.84) compared to the spinel counterpart, showing that the distribution of iron across the two sites was strongly modified by reduction post-treatment. Additionally, an increase of the mean spin canting angle to ca. 50° was also observed, indicating that the magnetic moments in this sample are less well-aligned relative to the external field, which also explains why the two subspectra are harder to separate.

The overlap of subspectra was even more pronounced for the Co/^57^Fe 32-red sample, and the more intense lines 2 and 5 clearly indicate the increased mean canting of ca 56° (Figure 3d), which is close to a random 3D orientation. Thus, this sample was moving toward an antiferromagnetic order, also evident by the disappearance of the hysteresis in the M(H) curves (Figure S5c). The inversion parameter, however, could not be calculated for this sample due to severe overlap of subspectra, and the data fit alone cannot discern them anymore. This was even more pronounced for the Co/^57^Fe 32 spinel sample, whereby the two subspectra could not be fitted anymore (Figure 3c).

All in all, the ethanol reduction post-treatment could change the electronic and crystal structure properties of the cobalt iron oxide spinel by forming a mixed phase of rock-salt and self-standing iron oxide moieties. ^57^Fe-enriched Mössbauer spectroscopy revealed the change of iron distribution in the sample toward an increasingly inverse spinel structure. This increase of the inversion parameter also hints toward the formation of magnetite (inverse spinel) clusters within the sample after the reduction process.

**Figure 4:**
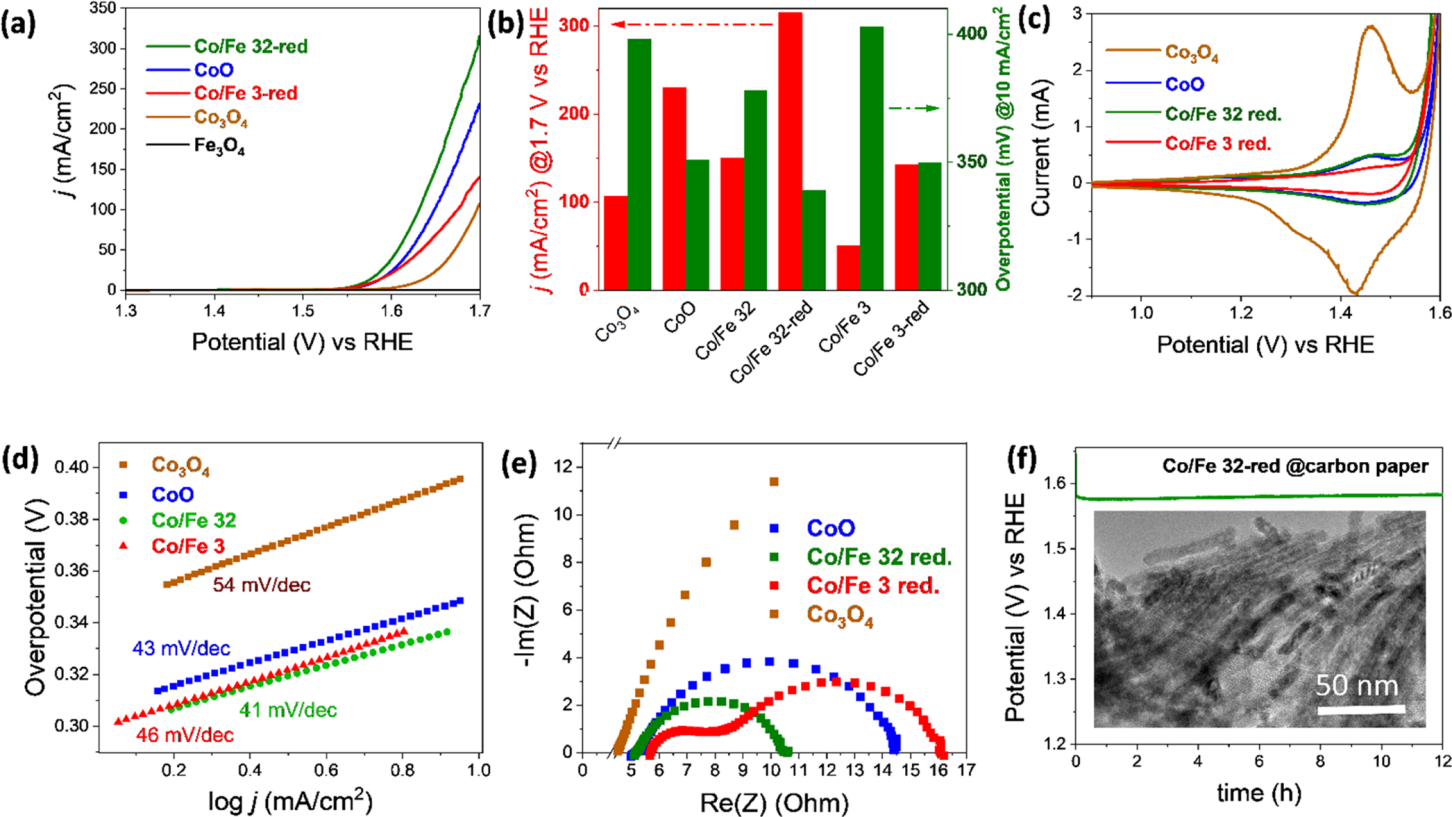
(a) OER LSV curves of reduced cobalt iron oxide in 1 M KOH solution compared to pristine Co_3_O_4_ and Fe_3_O_4_, (b) current density and overpotential summary of pristine cobalt iron oxide spinels and reduced cobalt iron oxide, (c) cyclic voltammogram comparison at 50th cycle at a scan rate of 50 mV/s, (d) kinetic measurement by a Tafel slope, (e) experimental Nyquist plot data, and (f) stability test measured at 10 mA/cm^2^ on carbon fiber paper with the corresponding TEM micrograph of the catalyst obtained from after 12 h of applied potential bias.

Following the detailed characterization, the OER catalytic activity of the ethanol-reduced samples was investigated in alkaline conditions using a 1 M KOH electrolyte. As shown by stabilized LSV curves (Figure 4a), CoO (reduced sample) showed much better OER activity and increased the reaction kinetics, as revealed by the Tafel slope (Figure 4d), compared to the pristine Co_3_O_4_. Moreover, the Co/Fe 32-red sample exhibited a higher current density at 1.7 vs RHE compared to CoO. In contrast, the activity decreased significantly when the iron content increased (Co/Fe 3-red). Further comparison with the pristine cobalt iron spinel series (Figure 4b) showed an overall twofold increase of current density at 1.7 V vs RHE for the reduced samples. A similar OER activity trend as in the spinel series is also observed on the iron oxide-supported CoO series. The overall increase of OER activity after reduction even for the samples with high iron content indicated the importance of rock-salt CoO formation to enhance the catalytic activity of the material. The Co/Fe 32-red sample had the optimal activity by decreasing the overpotential at 10 mA/cm^2^ from 378 to 339 mV, while the current density at 1.7 V vs RHE increased from 150 to 315 mA/cm^2^ compared to the spinel counterpart. This sample series showed a very good OER activity compared to the commercial RuO_2_ benchmark OER electrocatalyst (Figure S7) and recently published cobalt and cobalt iron-based electrocatalysts (Table S1).

Further observation of the initial stage and stabilized LSV curves (Figure S8a–d) showed a striking difference between spinel cobalt iron and reduced samples. There is no significant anodic peak change for the Co/Fe 32 spinel sample in the initial LSV and after stabilization with 50 cyclic voltammetry (CV) scan (Figure S8a). This indicated that the bulk spinel phase was retained after the stabilization. However, a clear change was readily observed on the Co/Fe 32-red sample even after the first LSV scan (Figure S8c). The broad and intense anodic peak at around 1.2 V vs RHE corresponding to Co^2+^ → Co^3+^ oxidation wave was significantly decreased after the first LSV scan.^30^ This intense anodic peak disappeared and was not observed in the second LSV scan. On the other hand, the Co^3+^ → Co^4+^ oxidation wave at around 1.45 V vs RHE became clearer after the stabilization. It gave the first hint of irreversible surface restructuring of Co^2+^ in the Co/Fe 32-red sample under applied potential bias. As shown by the CV curve (Figure S8b,d), Co/Fe 32-red had a more pronounced Co^2+^ → Co^3+^ oxidation wave (A1) compared to the spinel Co/Fe 32. Assuming the formation of a CoO*_x_*(OH)*_y_* intermediate state during potential bias, the increased intensity of the Co^2+^ → Co^3+^ oxidation wave in Co/Fe 32-red could be attributed to the formation of Co^2+^/^3+^O*_x_*(OH)*_y_*.^39,40^ The formation of Co^2+^ moieties in the intermediate state was proposed to be beneficial to initiate the formation of the μ-OO peroxide prior to the OER.^41^ Taking into account the Co^2+^/Co^3+^ as a descriptor for OER activity, Co^3+^ should be readily reduced into Co^2+^ in a reverse polarization scan. Hence, one can estimate the reducibility of Co^3+^ moieties as the second descriptor for OER activity. This Co^3+^-O reducibility was proposed to be estimated by comparing the current ratio on C1/C2 (Figure S8b,d).^39,42^

Although a small amount of magnetite clusters in Co/Fe 32-red could enhance the OER activity, a control experiment with mesoporous magnetite (Fe_3_O_4_) showed the lowest activity in the OER (Figure 4a). This pointed out the synergistic effect via the interaction of the magnetite cluster and CoO support to catalyze OER, which will be discussed further below in the *in situ* Raman and postmortem sections. Cyclic voltammetry (CV) was used to investigate the redox behavior of the selected samples. As seen in Figure 4c, a clear decrease of anodic peaks corresponding to Co^2+^ → Co^3+^ and Co^3+^ → Co^4+^ oxidation waves were observed for the Co/Fe 3-red sample. It goes in line with the decrease of OER activity of this sample compared to CoO. Hence, it can be proposed that the cobalt species are active centers for the OER. Furthermore, the introduction of iron oxide shifted the precatalytic, Co^2+^ → Co^3+^ oxidation wave to higher potential, which is proposed to disproportionate di-μ-oxo-bridged Co^3+^–Co^3+^ intermediates and resulted in altered electrokinetic behavior.^43^ A similar suppressing effect was also observed to take place in Ni^2+^ → Ni^3+^ oxidation in the presence of Fe.^44,45^

A kinetic investigation was further carried out using the Tafel slopes. As shown in Figure 4d, the calculated Tafel slopes are within a similar value (41–46 mV/dec) for the reduced sample, indicating that the OER mechanism was following the same reaction pathway and rate-determining step. These Tafel slope values are consistent with the previous work on the CoO*_x_* film by Dau et al., suggesting the single-site reaction mechanism with the oxidation of Co^3+^ → Co^4+^ as the rate-determining step.^30,43,46,47^ Among the reduced samples, Co/Fe 3-red has the slowest kinetics due to the insulating properties of the high iron content.^48^ Especially the Tafel slope values of reduced samples are lower than those of spinel cobalt iron oxide series (54–61 mV/dec), denoting that the iron oxide on CoO samples favored faster reaction kinetics compared to spinel counterparts.

Complementary electrochemical impedance spectroscopy (EIS) is utilized to probe the charge transfer behavior with the existence of iron oxide clusters after reduction. Figure 4e depicts the Nyquist plots, from which the charge transfer resistance could be calculated by fitting with the corresponding circuit models. For CoO and Co_3_O_4_, the simplified Randles model (*R*_Ω_)(*R*_ct_*Q*_dl_) was used to fit the experimental data, whereas (*R*_Ω_)(*R*_ct_*Q*_dl_)(*R*_film_*Q*_film_) was used to fit the data from Co/Fe 32-red and Co/Fe 3-red due to the existence of two semicircles in the Nyquist plot (Figure S9a–d).^49,50^ The fitted value is shown in Table S2. Likewise, the electrolyte resistance in this electrochemical cell was consistently measured at around 5 Ω for all samples. A pronounced decrease of charge transfer resistance (*R*_ct_) is observed on the sample with a small iron atomic ratio relative to cobalt (Co/Fe 32-red). This denotes the importance of synergistic effects by surface interaction on phase boundaries between iron oxide clusters with CoO support by facilitating charge transportation. The activity enhancement could only be observed at low iron content. With the increase of the iron content, additional semicircle, which can be related to the formation of less conductive oxide layer intermediate (*R*_film_), becomes more pronounced and hampers the conductivity. This could be originated from the formation of insulating FeOOH intermediate on Co/Fe 3-red during the OER.^48^ As a result, Co/Fe 3-red exhibits the slowest reaction kinetics even though it has a relatively similar overpotential compared to the CoO.

The electrochemical surface area (ECSA) of the samples was further calculated based on double-layer capacitance (*C*_dl_) measurements by a cyclic voltammetry method. Co_3_O_4_ has a higher *C*_dl_ (0.19 mF) compared to the rock-salt CoO (0.11 mF), which is likely due to the contribution of Co^3+^ in the spinel phase.^51,52^ It is analogous to the significant decrease of the *C*_dl_ value on the substitution of Co^3+^ by Fe^3+^ and Al^3+^ as reported by Behrens et al.^53^ Addition and further increase of the iron amount decrease the values of *C*_dl_ as well as ECSA (Figure S10a), denoting that the cobalt is the active species for the OER. However, the ECSA calculation by measuring *C*_dl_ in the non-Faradaic region might be complex and inaccurate due to the severe phase transformation of the rock-salt structure at elevated potential bias. The rock-salt phase itself serves as a precatalyst rather than the “real” catalyst. Taking into account these aspects and the impact of elemental composition on the *C*_dl_ value, a direct comparison of ECSA-normalized LSV curves might be an inaccurate descriptor.^53^ Hence, we use the BET surface area as the better descriptor of catalytic surface, as also suggested by Jaramillo et al. (Figure S10b).^54^ BET surface area-normalized LSV curves show that the specific activity of reduced cobalt oxide (CoO) is more superior compared to the pristine Co_3_O_4_. An optimum specific catalytic activity enhancement was observed with a small iron amount (Co/Fe 32-red).

The chronopotentiometry (CP) measurement is then performed by depositing the most active sample (Co/Fe 32-red) onto carbon fiber paper. This sample retains the activity at 10 mA/cm^2^ for up to 12 h (Figure 4f). A slight deactivation occurred due to the leaching of iron, as confirmed by ICP-OES by sampling the 1 M KOH electrolyte solution after the test (Table S3). The leached iron was calculated to be around 2 wt % of total iron contained in the deposited Co/Fe 32-red. The sample retained the nanowires’ morphology after prolonged chronopotentiometry tests without a visible formation of agglomeration, as seen in the TEM image.

The Faradaic efficiency (FE) for the molecular oxygen production over the catalysts before and after reduction was evaluated using an RRDE. For this purpose, as proof of the concept, samples of Co_3_O_4_ and Co/Fe 32 were chosen. Co_3_O_4_ and Co/Fe 32 electrocatalysts showed FE values of 94 and 96%, respectively, similar to the previously reported FE value of various cobalt oxide and cobalt iron oxides.^55,56^ The discrepancy from full electric charge utilization might be related to the alteration of the catalysts and side reactions like carbon oxidation.^57^ The carbon source for this side oxidation might come from the Nafion binder. However, this value is slightly decreased to 91 and 93%, respectively, after the reduction (Figure S11). This could be attributed to a drastic structural alteration of the reduced samples that consumes extra electrical charge during the applied potential, which will be discussed below in detail.

To gain insight into phase transformation and formation of intermediate phases upon applied potential, *in situ* Raman study, which is highly sensitive to probe the vibrational spectra of oxides, is then carried out in an *in situ* Raman electrochemical flow cell (Figure S12). The sample is deposited on the electrochemically roughened Au foil surface to enhance the sensitivity to probe local change on the catalyst surface via the surface-enhanced Raman spectra (SERS) effect.^11,30^ The CV and LSV curves recorded with the *in situ* Raman cell are shown in Figure S13a. The LSV curve shows that the blank roughened Au foil has negligible OER activity compared to the deposited sample within the potential range. Nevertheless, the roughened Au contributes to the evolution of an anodic peak at around 1.25 V vs RHE. To assign this anodic peak, a gradual 0.1 V increase of potential bias is applied, and the Raman spectra are recorded in each step (Figure S13b). This caused the formation of a small hump at 550 cm^–1^ starting from 1.3 V vs RHE that corresponds to Au–O stretching vibration.^31^ This peak is less intense compared to the one observed by Bell et al.,^31^ the possible cause for this discrepancy is that we used a 532 nm laser excitation wavelength that has weaker resonance properties to Au. Further oxidation on the Au foil does not increase the intensity of the observed hump. This vibration appears close to the potential windows of the evolved anodic peak; hence, the anodic peak observed on CV with blank roughened Au foil could be attributed to the oxidation of Au to AuO*_x_*. The anodic peak is slightly shifted to the negative potential with the sample due to the stabilization effect of the Au–CoOOH interaction that reduces the switching potential at which the oxidized phase is formed.^16^

**Figure 5:**
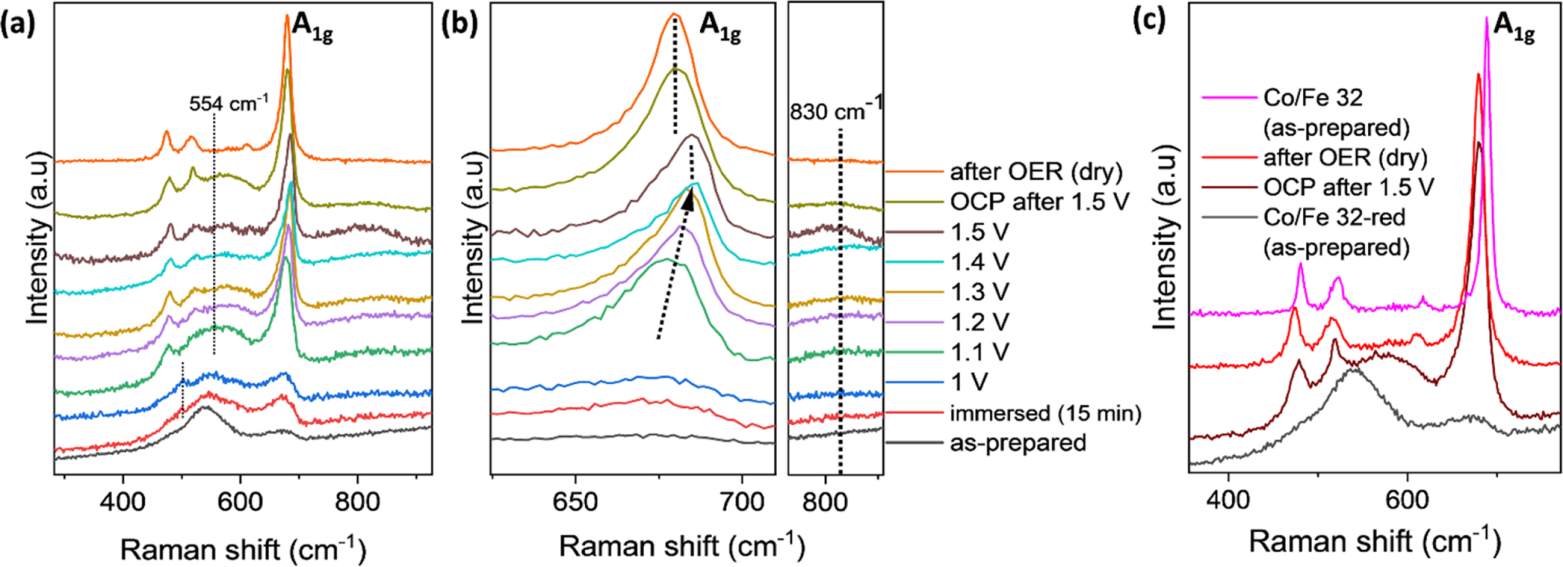
(a) *In situ* Raman spectra of the Co/Fe 32-red sample measured in a 1 M KOH electrolyte within a 0.1 V potential step. (b) Magnification of the A_1g_ band (left) and active oxygen species (right). (c) Spectra comparison of the Co/Fe 32-red sample measured by *in situ* Raman and the as-prepared Co/Fe 32 spinel before the reduction.

*In situ* Raman of the Co/Fe 32-red sample deposited on the roughened Au foil substrate revealed the evolution of the active intermediate state and irreversible phase transformation induced by applied potential bias in the OER (Figure 5a–c). The Raman spectra of the as-prepared sample show broad bands with two maxima at 535 and 680 cm^–1^ that correspond to CoO.^58^ The bands belonging to the iron oxide species are not visible due to the diluted amount of iron ratio compared to cobalt. Prior to the OER, the sample is immersed in 1 M KOH for 15 min to explore the impact of the solvation on the structural change on the catalyst’s surface. As seen in Figure 5a, the sample undergoes a surface restructuring upon 15 min of immersion in electrolyte solution without applied potential bias, denoting that the as-prepared sample acts as a precatalyst in this reaction. The band at 535 cm^–1^ becomes less intense, while the sharp band at 503 cm^–1^ and broad band at 520–620 cm^–1^, which are typical to the CoOOH, start to evolve.^59,60^ This indicates that the surface is reconstructed from rock-salt CoO (Co^2+^) to the oxyhydroxide (Co^3+^) structure.^61^ At the same time, a band at 680 cm^–1^ is red-shifted and gains more intensity. A relatively sharp peak at this region could also be attributed to the A_1g_ main band of the spinel phase;^7^ hence, the evolution of a minor amount of surface spinel phase upon immersion in 1 M KOH should be taken into account. The evolution of the Co(OH)_2_ phase is undetected due to the absence of a sharp peak at around 420 cm^–1^, that is originated from the A_1g_ vibration mode of Co(OH)_2_.^60^

The spectra remain unchanged at 1.0 V vs RHE applied potential bias; this indicates that the oxidation reactions on the catalyst’s surface have not taken place yet at this potential range. The observation verifies the absence of the anodic peak on CV at 1.0 V vs RHE (Figure S13a, inset). An anodic peak starts to evolve at 1.1 V vs RHE and goes inherently with the evolution of sharp bands at 477 and 675 cm^–1^ that could be attributed to F_2g_ and A_1g_ main bands of spinel, and a pronounced broad band centered at 554 cm^–1^ that corresponds to the A_1g_ vibrational mode of CoOOH. However, this peak is red-shifted compared to the center of the A_1g_ vibrational mode of CoOOH reported by previous studies and our control experiment with the mesostructured CoO catalyst (Figure S14d).^30,31^ One possible explanation for this discrepancy is the vibrational frequency decrease due to the bond elongation in the di-μ-oxo-bridged Co^3+^ O_h_ ions by the incorporation of Fe^3+^ (0.645 Å) with slightly longer ionic radii compared to Co^3+^ (0.610 Å).^39,62^ The iron might be originated from the Fe-oxyhydroxide structure (as a result of surface oxidation of small Fe_3_O_4_ clusters) with direct contact with CoOOH in the phase boundaries. Hence, this oxyhydroxide intermediate phase is further denoted as Co(Fe)OOH. This Co(Fe)OOH intermediate state is conductive under OER conditions and could enhance the charge transfer as shown by the aforementioned EIS measurements. In addition, the band corresponding to the rock-salt structure is weakly observed after 1.1 V vs RHE potential bias. Taking into account that Raman spectroscopy is a bulk sensitive technique and the high stability of spinel and oxyhydroxide phases in this potential range at pH 14 and 25 °C (based on Pourbaix diagram),^63,64^ the rock-salt phase transformation appears to occur not only on the surface but also in the bulk region. This will be further elaborated in the XRD section below. A similar phenomenon was also observed for the pristine CoO sample (Figure S14d,e). The control experiment with pristine Co_3_O_4_ (Figure S14a–c) indicated that the band corresponding to CoOOH is less pronounced compared to the one generated by the rock-salt counterpart. Since oxyhydroxide species is proposed to be the intermediate active phase for the OER, this discrepancy could also explain the lower activity of spinel to catalyze the OER compared to their rock-salt counterpart.^11,30,31^

Increasing potential bias up to 1.5 V vs RHE attenuates the intensity of the broad band centered at 554 cm^–1^ despite the inherent evolution of the band corresponding to Au–O vibration. This endorses that the Au–O vibration appears as a minor background, and the attribution of the band at this region is dominated by the Co(Fe)OOH phase. The attenuation of the band corresponding to the Co(Fe)OOH intermediate phase also indicates the further alteration of the catalyst into another intermediate phase. The direct interpretation of the shift of this peak for the formation of Co^4+^ could not be assigned due to its broad and lower intensity close to the OER onset potential.^31,65,66^

Nevertheless, the insight of the surface alteration close to the OER onset potential could be gained by following the trend on the A_1g_ band position and peak shape at around 675–690 cm^–1^ that corresponds to the Co^3+^–O vibration mode in the octahedral sites of spinel. As seen in Figure 5b, the A_1g_ band has asymmetric broadening. This asymmetric broadening supports the decrease of the crystallinity of the material due to the formation of amorphous surface species or randomizing effect of oxygen vacancies and surface bond contraction in the as-formed distorted spinel phase.^65,67^ In addition, the band is blue-shifted with the gradual increase of potential bias up to 1.4 V vs RHE. However, the peak location is unchanged on the OER onset potential (1.5 V vs RHE), presumably due to the already formed Co^4+^–O^•^ intermediate pre-equilibrium state as mechanism proposed by Hu et al.^30^ Blue-shifts in Raman bands indicate the lattice distortion due to the bond compression and charge redistribution.^65,66^ Considering the OER mechanism on cobalt oxide proposed by Hu et al.,^30^ the oxidation of six coordinated Co^3+^–O^•^ to Co^4+^–O^•^ is the potential determining step before the OER onset potential. Considering that the shorter atomic radii of six coordinated Co^4+^ are shorter than Co^3+^, this oxidation should shorten the overall bond length. This mechanism fits with our observation on the A_1g_ band, where the increasing potential bias up to the OER onset potential blue-shifts the A_1g_ band that can be correlated to the shortening of cobalt–oxygen bonding. This also goes in line with a slightly compressed Co–O bond that could still be observed in Co_3_O_4_ during the OER, indicating partial oxidation of the catalyst surface as reported by Liu et al.^41,68^ This finding is also consistent with the control experiment performed with the pristine CoO catalyst (Figure S14e). In contrast, the blue shift on the A_1g_ band was not observed in the control experiment with pristine Co_3_O_4_ (Figure S14c). The unshifted Raman band positions upon applied potential bias on Co_3_O_4_ under alkaline conditions were also reported in other works.^41,69^ The absence of the blue-shift, in this case, could be correlated to the defect-free spinel phase in the Co_3_O_4_ sample as shown by the symmetrical A_1g_ band compared to the asymmetric one generated from reduced CoO samples (Figure 5b). However, we cannot rule out the formation of the Co^4+^ intermediate in Co_3_O_4_ that could be simply undetected in this case due to the change that might take place only in several atomic layers that is under the limitation of *in situ* Raman’s instrument sensitivity. A pronounced phase transformation from Co_3_O_4_ to CoOOH, as reported by Yeo et al., was not observed here due to the relatively thicker powder catalyst deposition in our case compared to that work (0.4–87 monolayers).^31,69^

Additionally, a metastable peak at 830 cm^–1^ (Figure 5b) that exclusively evolves on the OER onset potential (1.5 V vs RHE) could be attributed to the “O–O” stretch of an adsorbed *O–OH species on the Au surface.^70−72^ Further investigation of this peak at higher potential was, however, limited by the severe bubble generation. To verify the assignment of this peak to the adsorbed *O–OH species on the Au surface, two more control experiments were then conducted with the roughened Au substrate in a 0.1 M KOH electrolyte to mitigate the bubble evolution and with the carbon fiber paper (CFP) substrate in 1 M KOH to eliminate the contribution of Au. For the first experiment with 0.1 M KOH, due to the lower activity in this diluted electrolyte, the impact of bubble evolution could be minimized and a good spectral resolution was obtained up to 1.65 V vs RHE (Figure S15). The phase transformation and peak shift were relatively more subtle in 0.1 M KOH compared to in 1 M KOH, possibly due to the weaker solvation impact in a more diluted electrolyte. Nevertheless, a similar shake-up peak at around 830 cm^–1^ could be observed at the visible onset potential of the OER in 0.1 M KOH (1.6–1.65 V vs RHE).

The second control experiment was carried out by depositing Co/Fe 32-red onto the CFP substrate and used as the working electrode in the *in situ* Raman cell. As shown in Figure S16a, the CFP substrate has no visible contribution to the overall measured current and OER activity. The *i*n*situ* measurement with the blank CFP substrate also shows no peak corresponding to the carbon oxidation within the region of interest (300–1200 cm^–1^). Spectra measured with the Co/Fe 32-red sample deposited on CFP (Figure S16c,d) reveals the absence of shake-up peak at around 830 cm^–1^, confirming that the observed peak at this location with the Au substrate was limited to the adsorbed *O–OH species on the Au surface. Besides, *in situ* Raman spectra measured in 1 M KOH with roughened Au and CFP substrates show an analogous trend of peak changes and peak shift, precluding the contribution of Au substrate in the interpretation of measured *in situ* Raman spectra.

By the time the applied potential is switched off, the A_1g_ band reverted to its original position. This indicates that Co^4+^–O^•^ is also metastable and governed by the potential bias. In addition, the broad peak centered at 554 cm^–1^ corresponding to the Co(Fe)OOH phase becomes more pronounced. It strengthens the hypothesis that the Co^4+^ is reduced back to the Co^3+^ state. The existence of an oxyhydroxide intermediate phase itself is highly influenced by the existence of the 1 M KOH electrolyte solution, as proven by the diminished Co(Fe)OOH band on the dry sample after the *in situ* tests (Figure 5a). Hence, this Co(Fe)–OOH intermediate might be the “real” catalyst for the OER. The phase transformation of rock-salt to the spinel phase after the *in situ* OER test is irreversible, as revealed by the change into the black color that is typical for the spinel color, in optical micrographs during the *in situ* Raman measurement and photographs of the as-prepared sample on the Au substrate and after the OER (Figure S17). In addition, this phase transformation induced by potential bias leads to the formation of a distorted spinel with a lower crystalline degree, as seen by the asymmetric peak broadening and shift on the A_1g_ band (Figure 5c).^73−75^ The overall geometry and structure of the material were maintained as observed from the chronopotentiometry test, eliminating the effect of phonon confinement to the change on Raman spectra.

**Figure 6:**
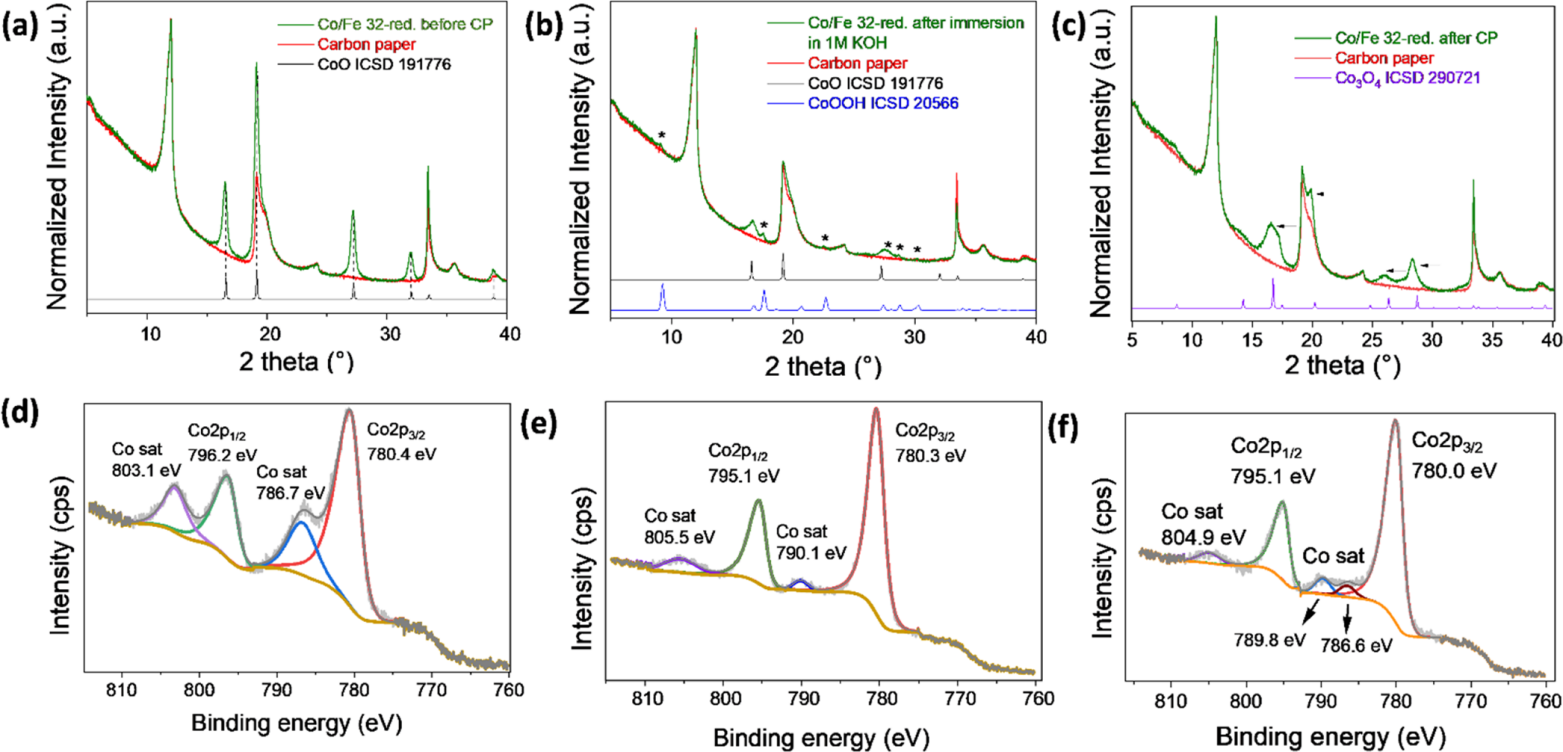
XRD patterns of Co/Fe 32-red (a) before CP, (b) after 12 h immersion in 1 M KOH, and (c) after 12 h of CP. The XRD data was measured on carbon fiber paper in a transmission mode with a Mo Kα_1_ (λ: 0.7093 Å) radiation source. High-resolution Co 2p XPS data of Co/Fe 32-red are shown in (d) before CP, (e) after 12 h immersion in 1 M KOH, and (f) after 12 h of CP.

In-depth postmortem analysis was then conducted to characterize the catalyst after the OER, where carbon fiber paper is used as a working electrode. First, the Co/Fe 32-red sample deposited on carbon fiber paper was immersed in 1 M KOH for 12 h to verify the solvation impact on the material. Under the alkaline conditions, the surface reconstruction took place even without the applied potential bias as confirmed by XRD (Figure 6a,b). The emerged CoOOH phase (and potentially Co(Fe)OOH), as also observed from *in situ* Raman, indicated that the octahedrally coordinated Co^2+^ ions at the surface were partly oxidized into some octahedrally coordinated Co^3+^ ions where CoO is still detectable.

This finding was further supported by high-resolution Co 2p XPS, where Co^3+^ from CoOOH was predominantly found on the surface upon solvation in KOH, compared to only Co^2+^ from CoO before CP (Figure 6d,e). The assignment of Co^2+^ is based on the intense shake-up satellite observed around 786 eV, while Co^3+^ exhibits a weak satellite around 790 eV.^76−78^ Next, the rock-salt-type CoO phase was transformed into the Co_3_O_4_ spinel structure after prolonged potential bias (Figure 6c,f). This goes in line with the previously diminished Co^2+^ → Co^3+^ oxidation wave even after the first LSV scan and observation from *in situ* Raman, indicating that, unlike its spinel counterpart, CoO transformed irreversibly upon potential bias. A shift of the spinel reflections to smaller 2θ angles could be attributed to the contribution of the thickness of carbon paper, which violates the focusing conditions of the diffraction event, or to some lattice disorder due to the presence of iron in the electrochemically formed spinel phase.^39^ HR-TEM images of the sample material removed from the working electrode showed the formation of an amorphous phase on the surface of the nanowires (Figure S18a). This amorphous phase was an artifact of surface restructuring and the formation of amorphous Co(Fe)OOH as an intermediate state during applied potential bias as observed in the *in situ* Raman.^6,23^ Further magnification of the nanowires and nanoparticle interface (Figure S18b) reveals that the phase boundaries between the nanowire matrix and nanoparticle disappeared after the OER and formed a singular crystalline spinel structure. This verifies that the formation of the conductive Co(Fe)OOH intermediate phase during applied potential bias is governed by the direct contact between the iron oxide cluster with the cobalt oxide nanowire matrix in the phase boundaries.

Finally, SEM-EDX measurements were carried out to verify the iron leaching (Figure S18c,d). The increasing atomic ratio of Co/Fe after 12 h of CP indicates that some of the iron was etched into the electrolyte, as also confirmed by our ICP-OES analysis (Table S3). The in-depth *in situ* and postmortem characterization suggested that the iron oxide clusters supported on CoO nanowires acted as the precatalyst for the OER. Under applied potential bias, the reduced sample undergoes a phase transformation into oxyhydroxide intermediates and a distorted spinel phase. The distorted spinel phase from this transformation is beneficial to enhance the OER activity of the sample. Kinetic and EIS studies further revealed that cobalt moieties play a role as the active sites for the OER, whereas the iron oxide clusters were beneficial to enhance the electronic charge transfer. However, a high amount of iron is detrimental to the OER activity due to the formation of FeOOH insulating species.

## Conclusions

A highly active OER catalyst has been prepared through a mild reduction of cobalt iron oxide spinel nanowires. Following the reduction, iron oxide clusters supported by the ordered mesoporous CoO nanowire matrix were generated. A significant change in an ionic distribution upon reduction could be confirmed by Mössbauer spectroscopy, thanks to the enrichment of powder samples with the ^57^Fe isotope. The reduction post-treatment improved the OER performance significantly by boosting the current density from 150 to 315 mA/cm^2^ at 1.7 V vs RHE and lowering the overpotential down from 378 to 339 mV at 10 mA/cm^2^. *In situ* Raman and postmortem studies revealed that the reduced material acted as the precatalyst for the OER due to the transformation of rock-salt CoO into disordered cobalt oxide spinel through the implementation of external potential bias. It has been found that Co^2+^ in the intermediate step of the reduced samples was more pronounced compared to the spinel counterpart. This could enhance the formation of the μ-OO peroxide prior to the OER as well as the Co^3+^-O reducibility in the reverse scan as a key activity descriptor. The interaction of iron clusters with CoO at the phase boundaries was found to be lucrative for the formation of more active OER species.

## Supporting Information Available

Textural parameters of samples before and after reduction; TEM micrographs of samples before reduction; STEM micrographs of samples after reduction; structural characterization data of the ^57^Fe-enriched samples; magnetometry data of ^57^Fe-enriched samples; Mössbauer spectra of reference cobalt iron oxide; LSV curves compared to the RuO_2_ benchmark catalyst; table of OER performance reported by analogous works; CV profiles of samples before and after reduction; EIS fitting and the equivalent circuit model; table of fitted EIS data; table of electrolyte solution’s ICP-OES data; double-layer capacitance measurement; BET normalized LSV curves; Faradaic efficiency measurement of samples before and after reduction; photograph and schematic view of *in situ* Raman electrochemical cell; LSV and CV curves measured in the *in situ* Raman cell; *in situ* Raman data of the blank Au substrate; *in situ* Raman data of Co_3_O_4_ and CoO; *in situ* Raman data measured in 0.1 M KOH; *in situ* Raman data measured with the carbon fiber paper (CFP) substrate; optical micrographs recorded during the *in situ* Raman acquisition; postmortem data characterization of Co/Fe 32-red (PDF)
