# Supplementary material for: Phase Segregation in Cobalt Iron Oxide Nanowires toward Enhanced Oxygen Evolution Reaction Activity
Source: JACS Au. 2022 Feb 25;2(3):697–710. doi: 10.1021/jacsau.1c00561 (PMC8970005; doi:10.1021/jacsau.1c00561)
Supplement: Supplementary file 1 — au1c00561_si_001.pdf [file au1c00561_si_001.pdf]

Supporting Information

Phase Segregation in Cobalt Iron Oxide Nanowires toward Enhanced Oxygen Evolution Reaction Activity

Eko Budiyanto,[#] Soma Salamon,[§] Yue Wang,[#] Heiko Wende,[§] and Harun Tüysüz*[#]

Max-Planck-Institut für Kohlenforschung, Kaiser-Wilhelm-Platz 1, 45470 Mülheim an der Ruhr, Germany

§ Faculty of Physics and Center for Nanointegration Duisburg-Essen (CENIDE), University of Duisburg-Essen, 47057 Duisburg, Germany

*E-mail: tueysuez@kofo.mpg.de

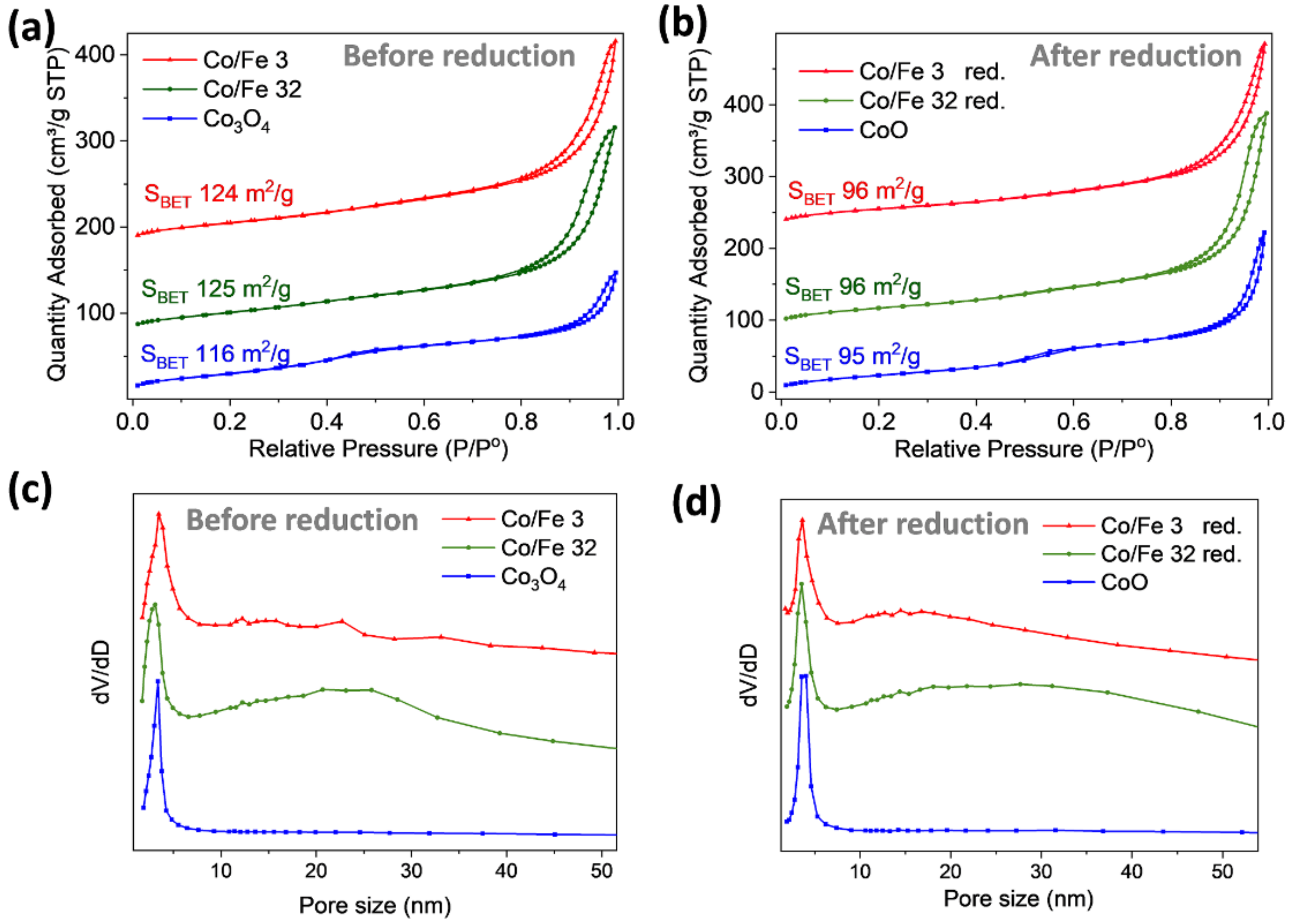

Figure S1. Nitrogen physisorption data of cobalt iron oxide. (a) Adsorption-desorption hysteresis loop of sample series before reduction and (b) after reduction. Pore size distribution of the sample series (c) before reduction and (d) after reduction. The pore size distribution was calculated by Barrett, Joyner, and Halenda (BJH) method from the desorption branch.

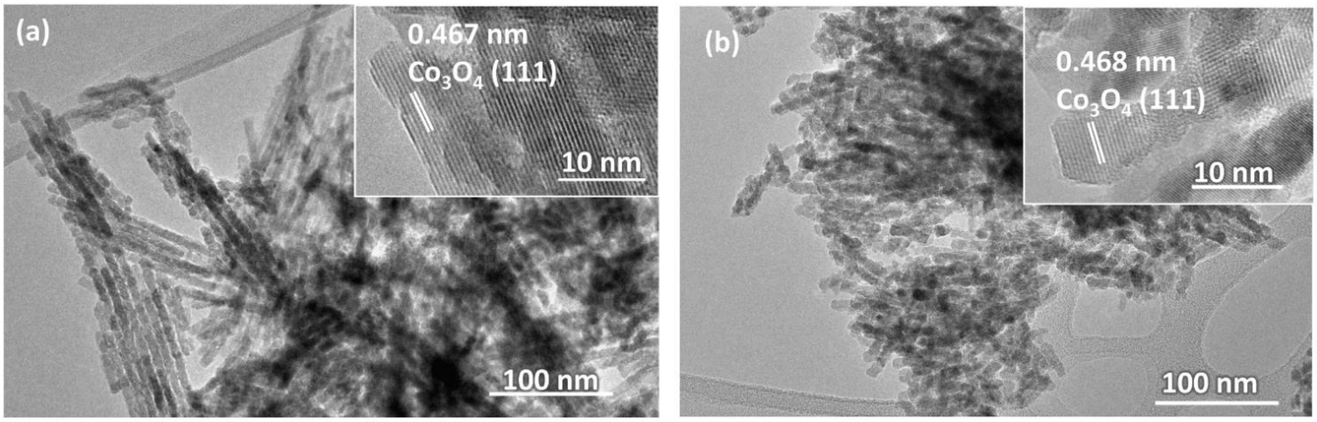

Figure S2. TEM micrographs of samples before reduction (a) Co/Fe 32, inset: HR-TEM, (b) Co/Fe 3, inset: HR-TEM.

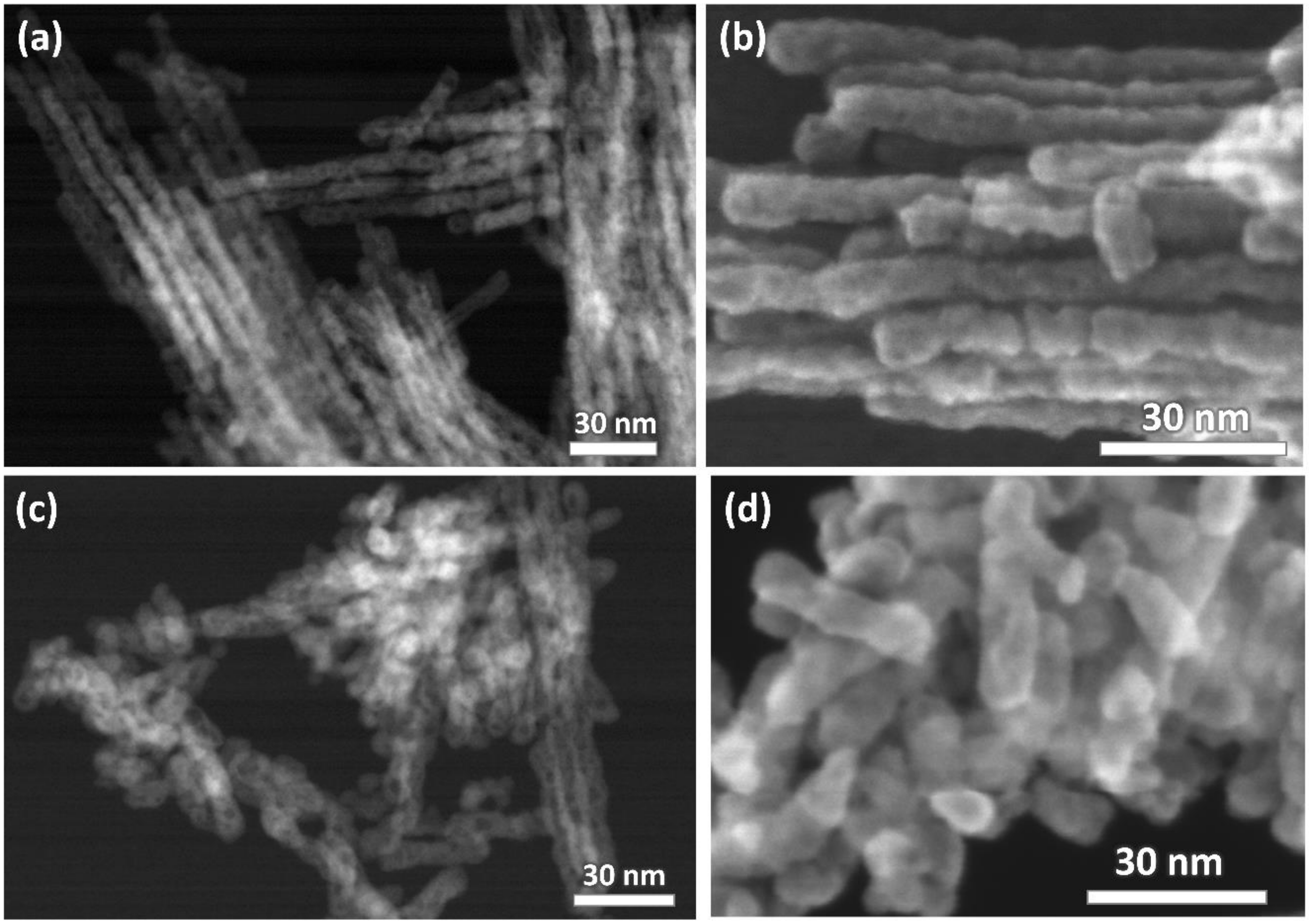

Figure S3. (a) Dark-field and (b) secondary electron imaging mode of Co/Fe 32-red. (c) Dark-field and (d) secondary electron imaging mode of Co/Fe 3-red.

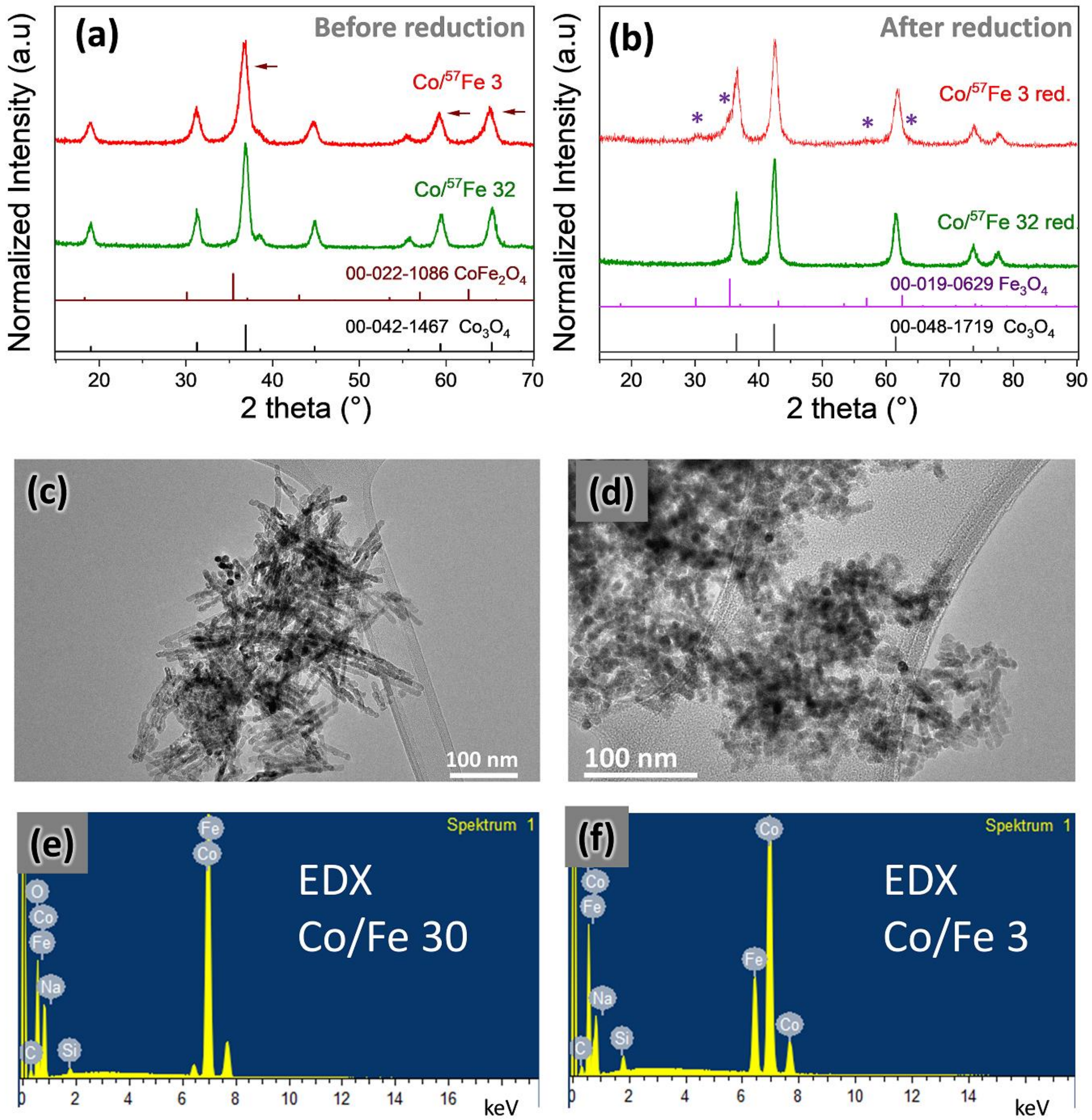

Figure S4. (a) Powder XRD reflectances of isotope-labeled Co/^{57}Fe sample before reduction and (b) after reduction. The XRD data was measured with Cu K$\alpha_{1,2}$ radiation X-ray source (λ: 1.5406 Å). TEM images of (c) Co/^{57}Fe 32-red and (d) Co/^{57}Fe 3-red. Bulk EDX spectra of (e) Co/^{57}Fe 32-red and (f) Co/^{57}Fe 3-red.

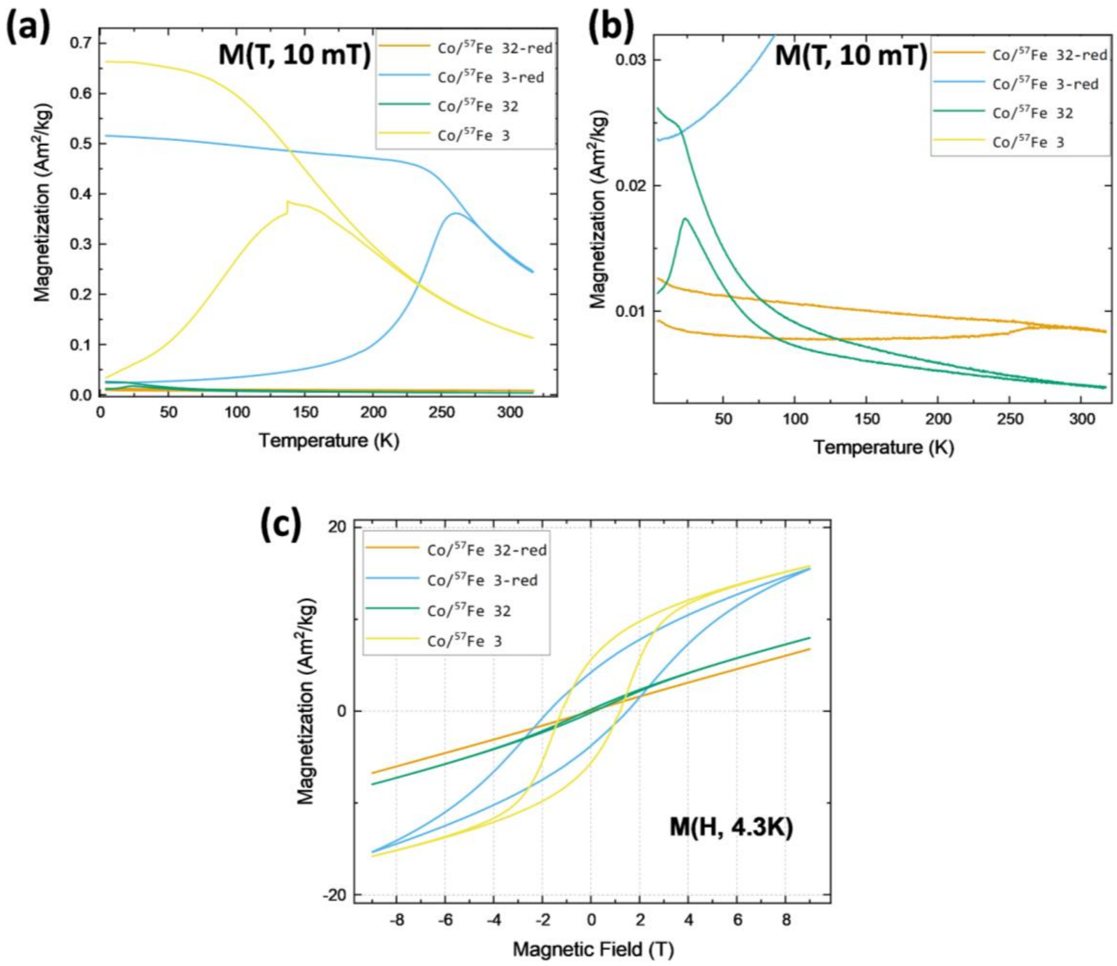

Figure S5. (a) Zero field cooled-field cooled (ZFC/FC) temperature-dependent magnetization/M(T) curve measured at 10 mT with (b) magnification of low iron content curves. (c) Magnetic field-dependent magnetization/M(H) curves measured at 4.3 K.

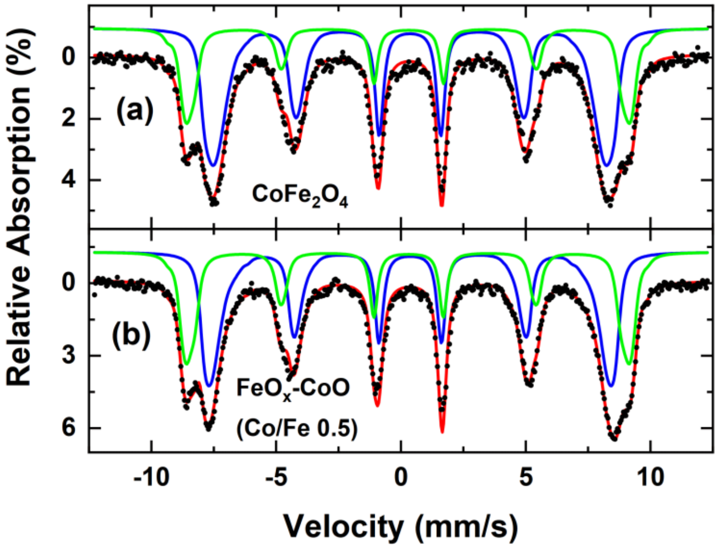

Figure S6. Reference spectra for (a) $CoFe_2O_4$ and (b) FeO_x-CoO (Co/Fe 0.5) were recorded at 4.3 K with an applied field of 5 T relative to the γ-ray propagation direction. The subspectra correspond to tetrahedrally coordinated A-sites (green) and octahedral B-sites (blue).

Table S1. OER performance of cobalt and cobalt iron-based electrocatalysts reported in analogous works.

Catalyst	S_{BET} (m^2/g)	Electrolyte	Calculated loading (mg/cm^2)	Tafel slope (mV/dec)	η (mV) vs RHE at 10 mA/cm^2	Reference
Fe_3O_4-CoO nanowires (Co/Fe 32-red)	96	1 M KOH	0.12	41	339	This work
$Co_{3-x}Fe_xO_4$ (Co/Fe 32) nanowires	125	1 M KOH	0.12	55	378	1
Electrodeposited CoO_x	n.a.	1 M NaOH	n.a.	n.a.	390	[2]
Co_3O_4 coffee-templated	55	1 M KOH	0.12	59	400	[3]
n-Co_3O_4 (more Co^{3+} reducibility)	136	1 M KOH	50 μg (glassy carbon surface area is not stated)	153	380	[4]
c- Co_3O_4 (higher oxygen defects)	29	1 M KOH	50 μg (glassy carbon surface area is not stated)	53	440	[4]
Co_3O_4	n.a.	1 M KOH	0.69	143	251 at 40 mA/cm^2	[5]
Electrodeposited $CoFeO_x$	n.a.	1 M NaOH	n.a.	n.a.	370	[2]
$Co_{3-x}Fe_xO_4$ (Co/Fe 32) KIT-6 templated	102	0.1 M KOH	0.12	n.a.	486	[6]
Co_3Fe_7 alloy	n.a	0.1 M KOH	1.97	70.8	440	[7]
Commercial $CoFe_2O_4$	32	1 M KOH	0.20	95	416	[8]
Commercial $CoFe_2O_4$ (50 passages laser fragmentation)	n.a.	1 M KOH	0.20	71	320	[8]
Fe_2O_3 (< 5 μm)	n.a	1 M NaOH	n.a	n.a	1230	[9]
Co–Fe double-atom catalyst	n.a	1 M KOH	0.33	44	360	[10]
Co–Fe double-atom catalyst on carbon cloth	n.a	1 M KOH	n.a	40	321	[10]
Fe adsorbed CoOx	n.a	1 M KOH	n.a	28	309	[11]

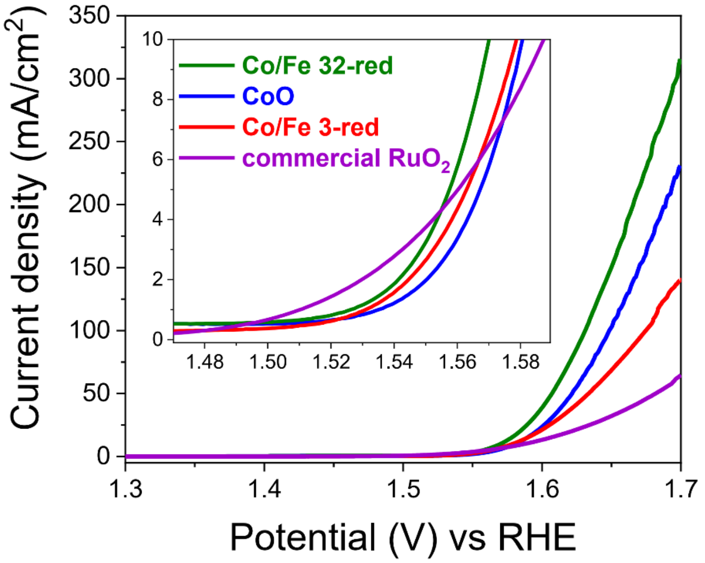

Figure S7. LSV curves of samples in this work measured after 50th CV compared to the commercial RuO_2 catalyst.
RuO_2 powder was purchased from Sigma-Aldrich, 99.9% trace metals basis, CAS No. 12036-10-1. RuO2 activity was observed to be deactivated under a harsh oxidation condition in 1M KOH.[12]

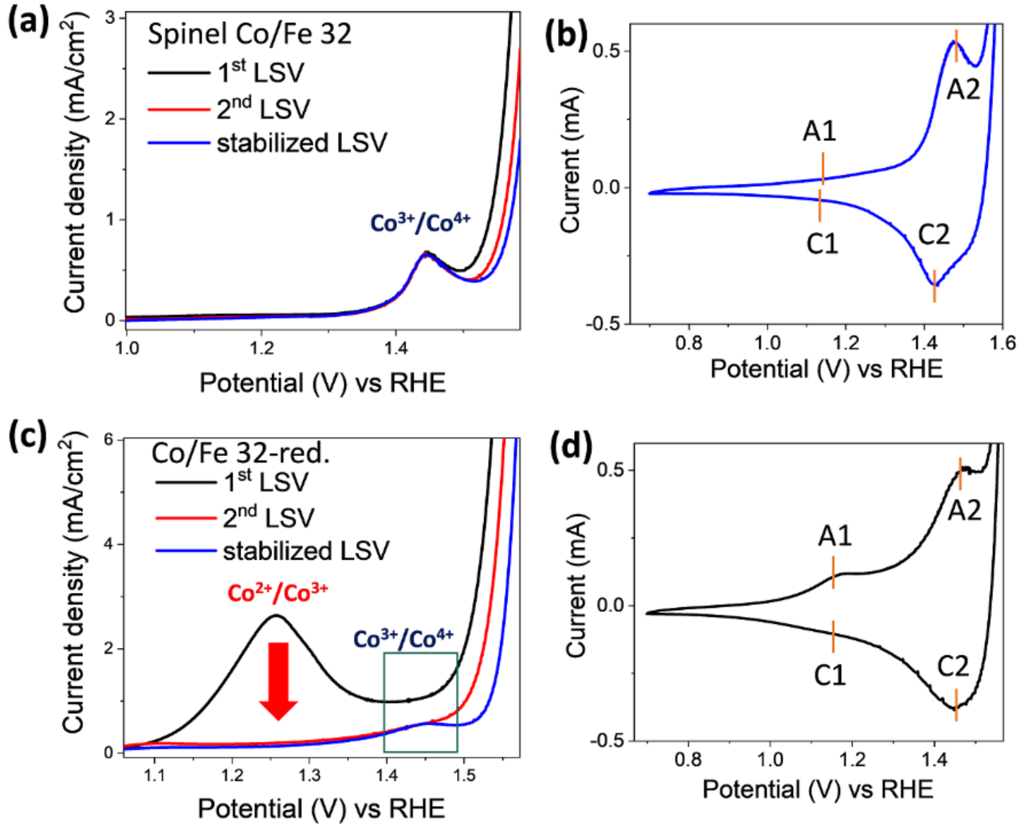

Figure S8. (a) LSV profile and (b) cyclic voltammogram of spinel Co/Fe 32. (c) LSV profile and (d) cyclic voltammogram of Co/Fe 32-red. A1 and A2 denote the anodic peaks, C1 and C2 denote the cathodic peak.

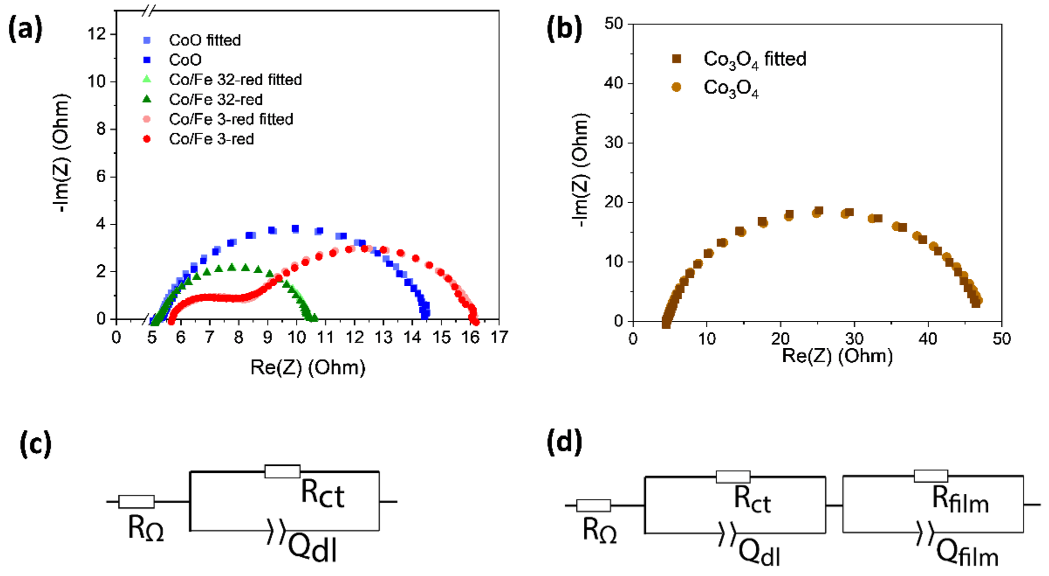

Figure S9. Nyquist plots fitted with the corresponding circuit models of (a) reduced samples, (b) Co_3O_4. (c) Randles circuit $(R_\Omega)(R_{ct}Q_{dl})$, and (d) equivalent circuit model for two semicircles $(R_\Omega)(R_{ct}Q_{dl})(R_{film}Q_{film})$.

The constant phase element (Q) is used instead of capacitance (C) to fit the Nyquist plot to compromise the deviation from ideal capacitance.[13, 14] Symbols: electrolyte resistance (R_Ω), constant phase element of the double layer (Q_{dl}), constant phase element of the inner oxide layer (Q_{film}), charge transfer resistance (R_{ct}), and inner oxide layer resistance (R_{film})

Table S2. EIS data fitting.

	R_Ω (Ω)	R_{film} (Ω)	R_{ct} (Ω)
Co_3O_4	4.5	-	43.1
CoO	5.3	-	9.3
Co/Fe 32-red	5.1	0.3	5.0
Co/Fe 3-red	5.5	2.9	7.7

Table S3. ICP-OES data of electrolyte solution

Sample	Co (ng/g)	Fe (ng/g)
Before stability test	<2.4	<2.4
After stability test	<2.4	5.1

The sample was 1M KOH electrolyte taken before and after the stability test with Co/Fe 32-red catalyst.

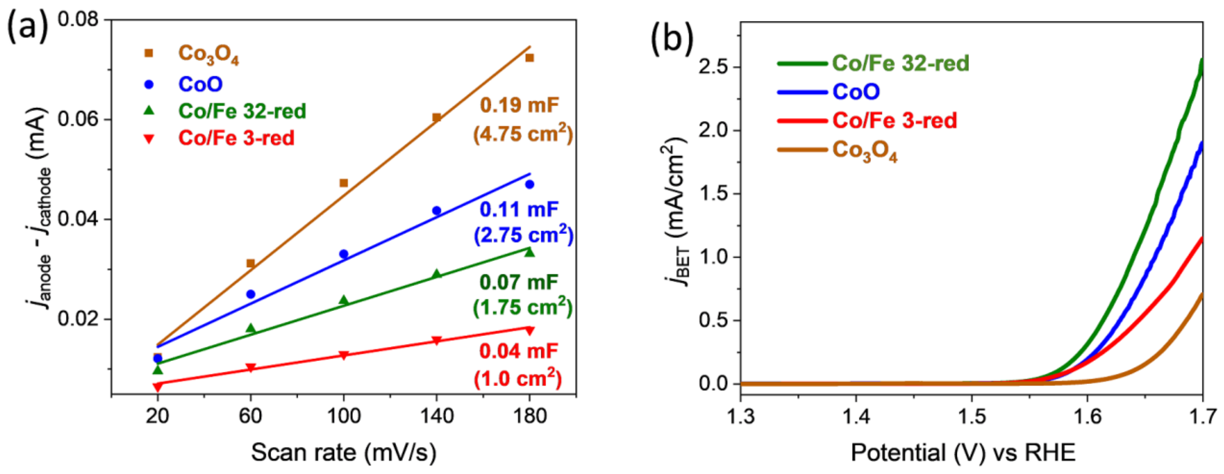

Figure S10. (a) Capacitive current differences ($\Delta j = j$ anode − j cathode) collected at 1.05 V vs RHE with increasing scan rate, the value inside the bracket is electrochemical surface area (ECSA) value. (b) BET surface area-normalized LSV curves.

Electrochemical double-layer capacitance (C_{dl}) measurements were carried out with cyclic voltammetry (CV) method within 0.1 V potential range at non-Faradaic potential with varying scan rates (20, 60, 100, 140, and 180 mV/s).[2] C_{dl} values were calculated as the half slope of the scan rate vs capacitive current differences. The electrochemical surface area (ECSA) was calculated from the obtained C_{dl} value with the equation: ECSA = C_{dl}/Cs, where Cs is the specific capacitance. 0.04 mF/cm^2 is used as the reference value of the catalysts for OER in 1 M KOH.[2]

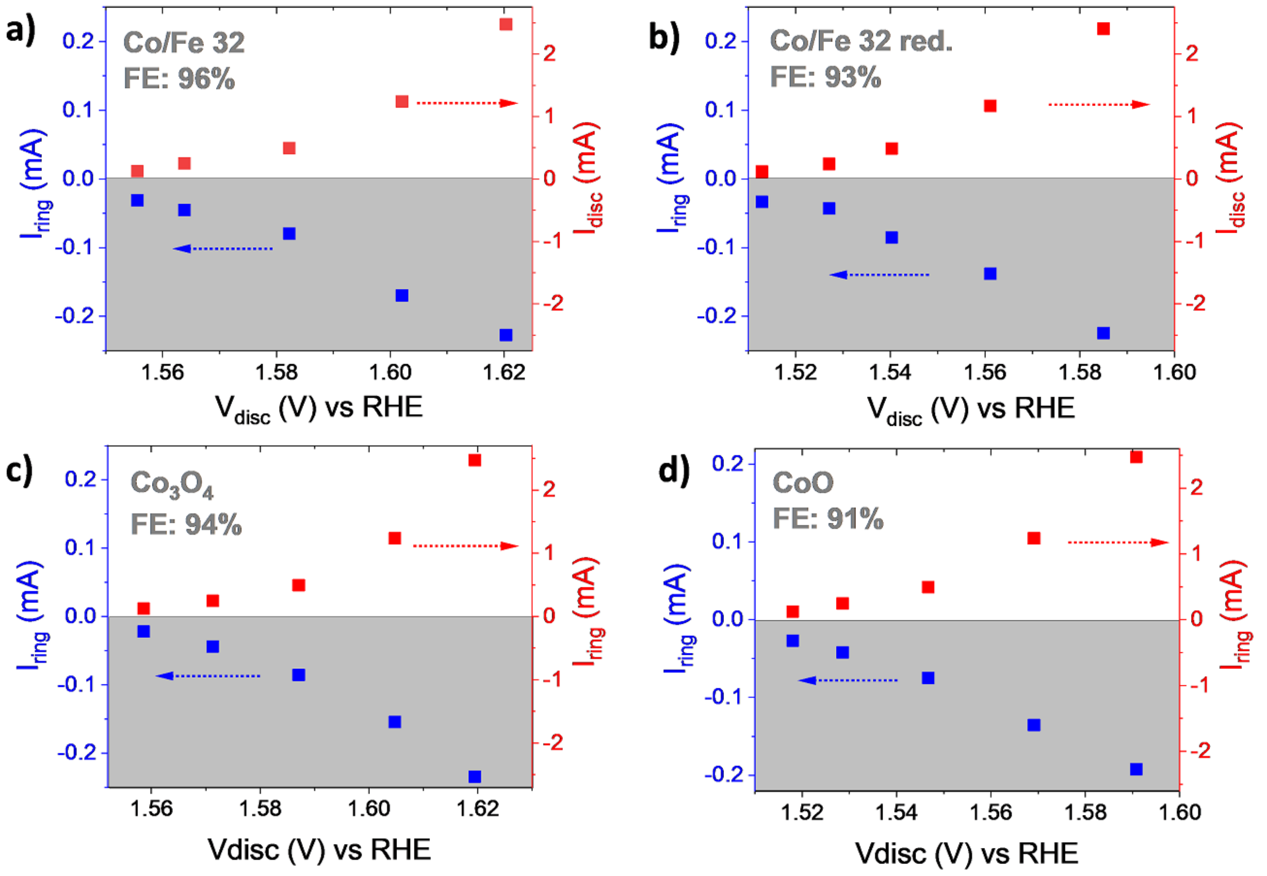

Figure S11. Faradaic efficiency measurement of (a) Co/Fe 32, (b) Co/Fe 32-red, (c) Co_3O_4, and (d) CoO that shows O_2 production.

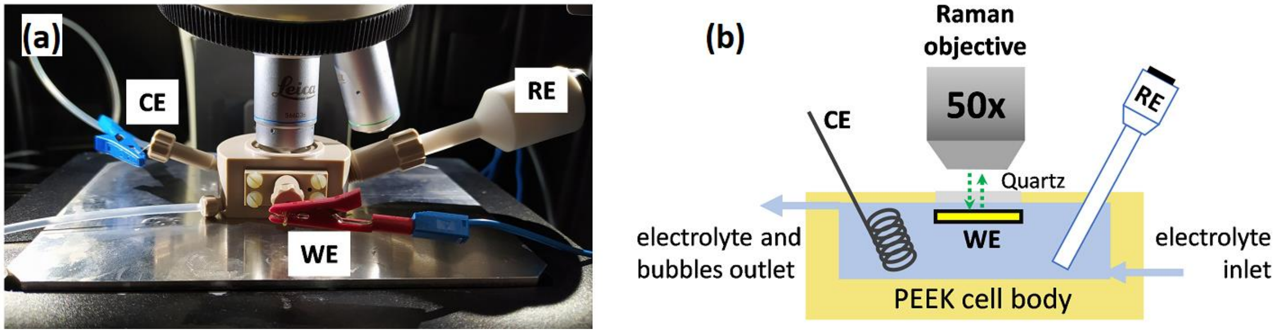

Figure S12. (a) Photograph of custom-made *in situ* Raman electrochemical flow cell and its (b) schematic illustration. RE: reference electrode, WE: working electrode, CE: counter electrode.

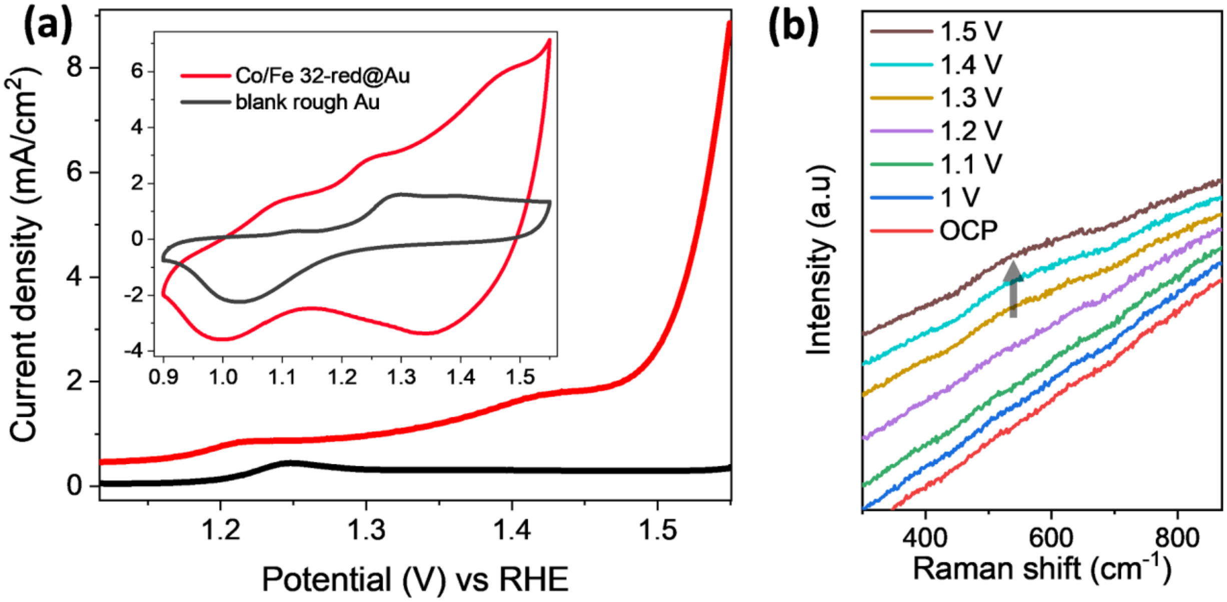

Figure S13. (a) LSV curve and CV (inset) of blank rough Au foil and sample deposited on rough Au foil measured with the *in situ* Raman cell. (b) *in situ* Raman spectra of blank rough Au foil.

The *in situ* Raman measurements were carried out in a chronoamperometric (CA) mode by holding potential for 1 minute. The as shown LSV and CV curves serve a purpose for visualization of OER reaction in the *in situ* Raman cell as well as to shed a light on the possible potential range where the surface alteration occurs.

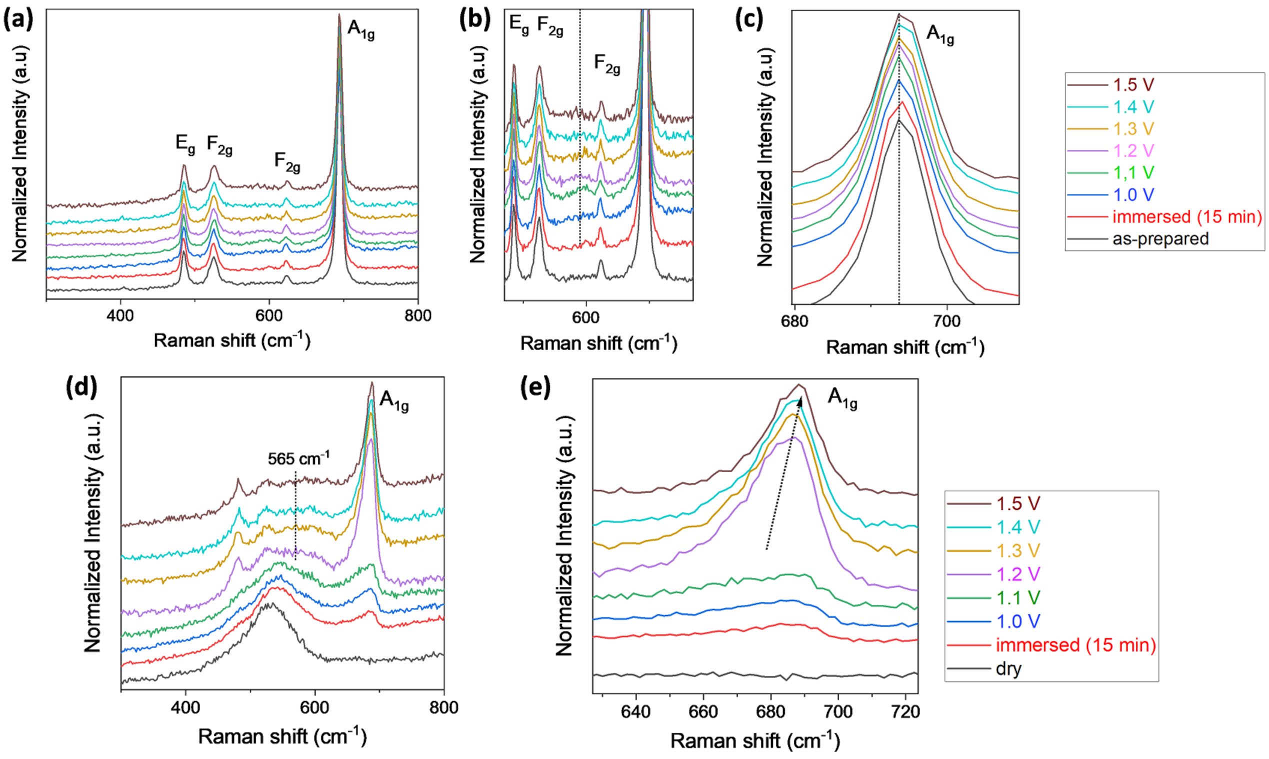

Figure S14. (a) *In situ* Raman spectra of Co_3O_4 sample measured in 1M KOH electrolyte within 0.1 V potential step, (b) magnification of the band corresponding to oxyhydroxide species and (c) A_{1g} band. (d) *In situ* Raman spectra of CoO sample measured in 1M KOH electrolyte within 0.1 V potential step and (e) magnification on its A_{1g} band. The legends are given on the right side of the respective sample figures.

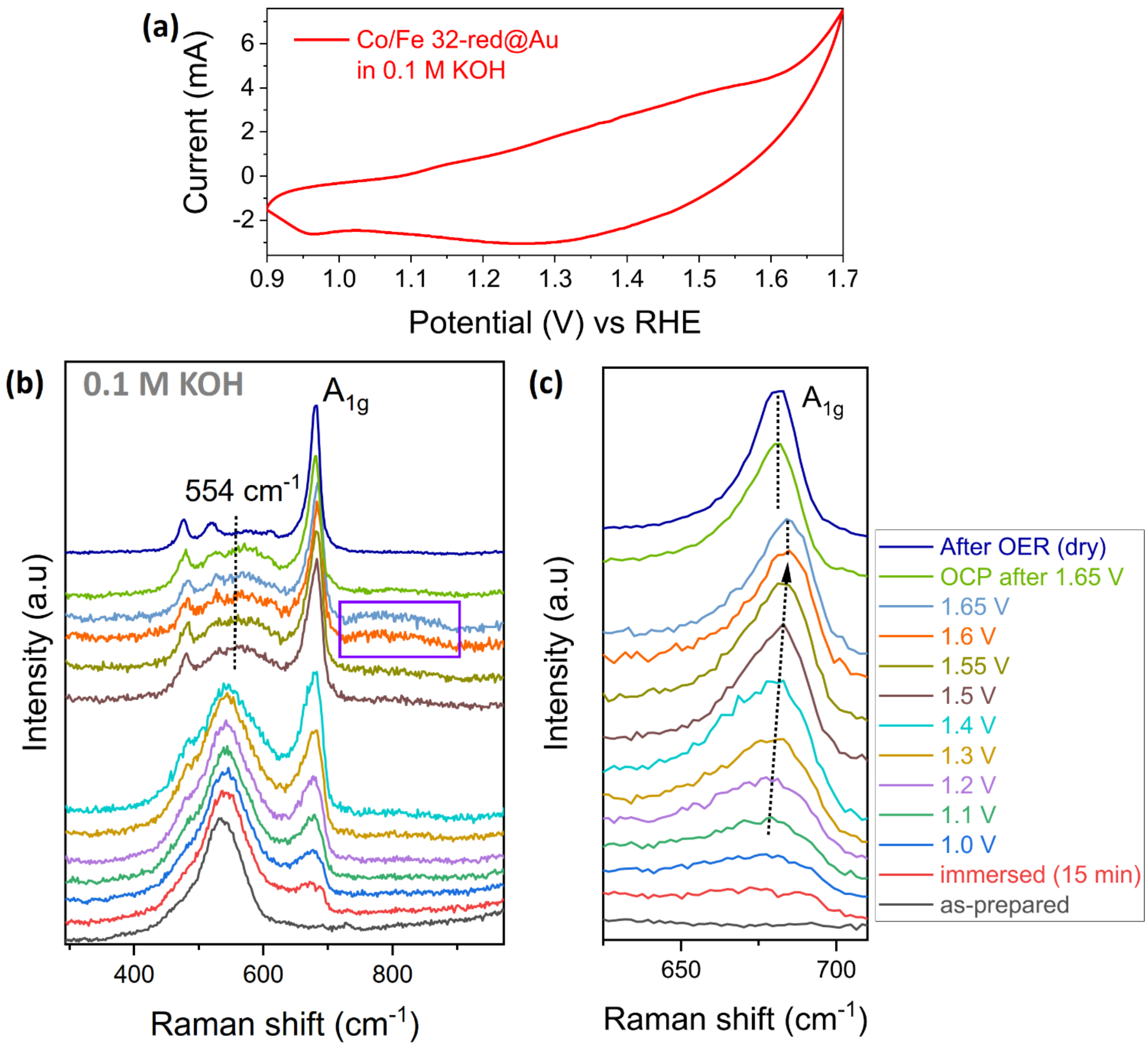

Figure S15. (a) CV curve measured in 0.1 M KOH inside the *in situ* Raman cell. (b) *In situ* Raman spectra of Co/Fe 32-red sample deposited on roughened Au substrate measured in 0.1 M KOH electrolyte, (c) magnification on its A_{1g} band. The purple box indicates the evolution of peak corresponding to the adsorbed *O-OH species on Au surface.

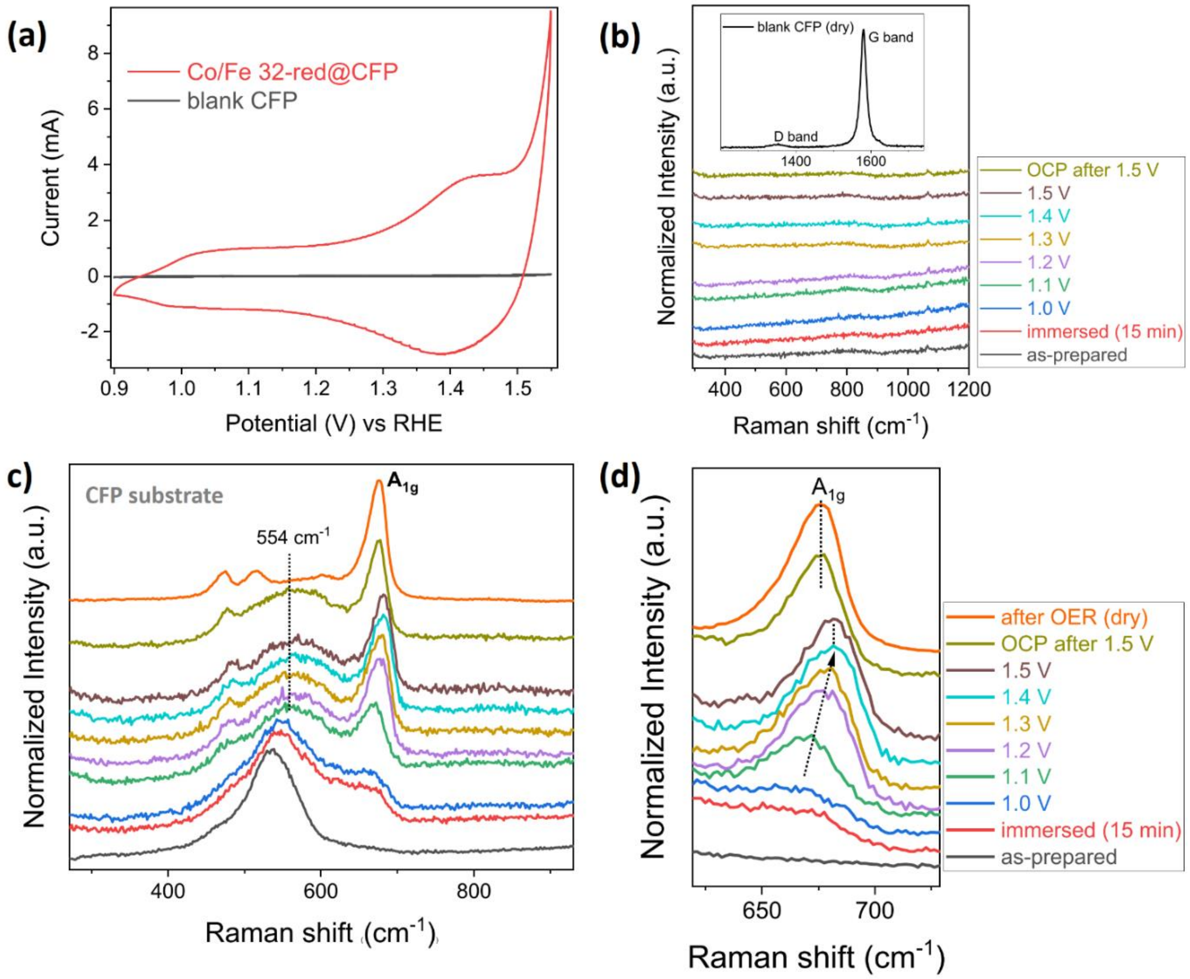

Figure S16. (a) Cyclic voltammetry recorded in the *in situ* Raman cell and (b) *In situ* Raman spectra of blank CFP (carbon fiber paper). CFP contains a graphitic phase as shown by the stark G band that corresponding to sp^2 configuration. (c) *In situ* Raman spectra of Co/Fe 32-red sample deposited on the CFP substrate, (d) magnification on its A_{1g} band. The measurements were carried out in 1 M KOH electrolyte.

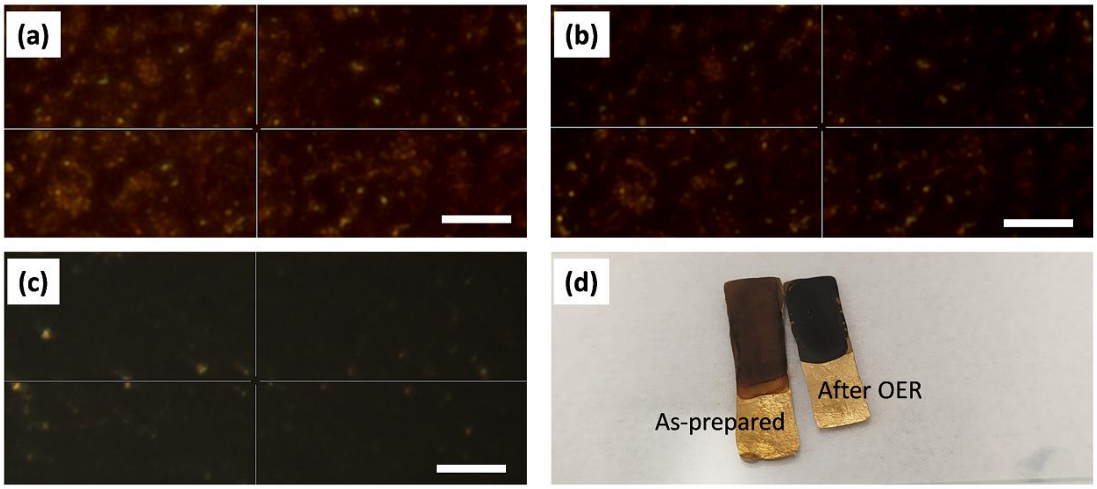

Figure S17. Optical micrographs during *in situ* Raman measurement recorded during (a) immersion in 1M KOH, (b) after 1.2 V vs RHE, and (c) after OER. (d) Photograph comparing the color change of the as-prepared sample and after OER. The scale bars of the optical micrographs are 20 μm.

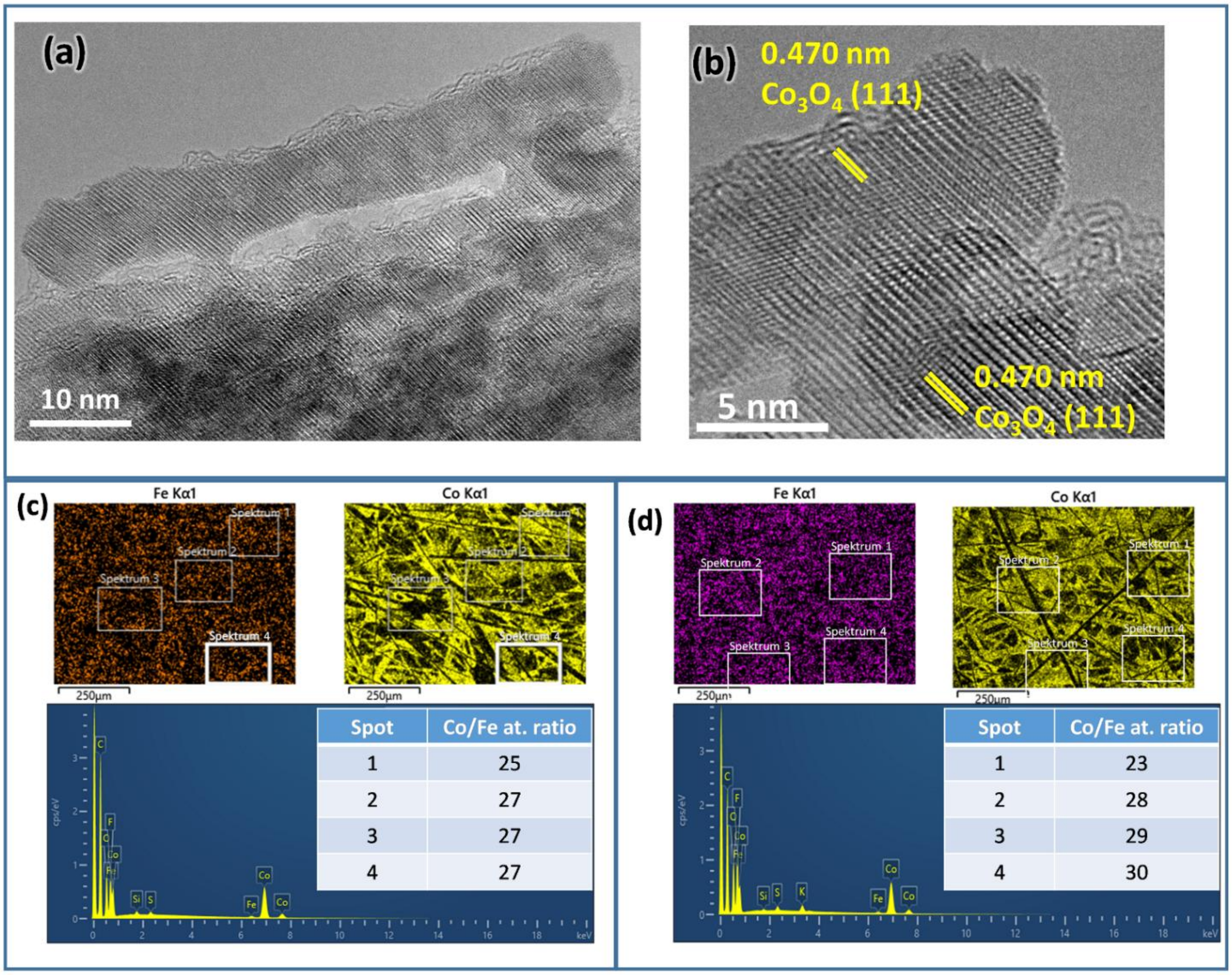

Figure S18. (a-b) HR-TEM images of Co/Fe 32-red after 12 h of CP. SEM-EDX mapping and spot elemental analysis of Co/Fe 32-red (c) before CP and (d) after 12 h of CP.

References:

1. Budiyanto, E.; Yu, M.; Chen, M.; DeBeer, S.; Rüdiger, O.; Tüysüz, H., Tailoring Morphology and Electronic Structure of Cobalt Iron Oxide Nanowires for Electrochemical Oxygen Evolution Reaction. *ACS Appl. Energy Mater.* **2020,** *3* (9), 8583-8594.
2. McCrory, C. C. L.; Jung, S.; Peters, J. C.; Jaramillo, T. F., Benchmarking Heterogeneous Electrocatalysts for the Oxygen Evolution Reaction. *J. Am. Chem. Soc.* **2013,** *135* (45), 16977-16987.
3. Yu, M.; Chan, C. K.; Tüysüz, H., Coffee-Waste Templating of Metal Ion-Substituted Cobalt Oxides for the Oxygen Evolution Reaction. *ChemSusChem* **2018,** *11* (3), 605-611.
4. Alex, C.; Sarma, S. C.; Peter, S. C.; John, N. S., Competing Effect of Co^{3+} Reducibility and Oxygen-Deficient Defects Toward High Oxygen Evolution Activity in Co_3O_4 Systems in Alkaline Medium. *ACS Appl. Energy Mater.* **2020,** *3* (6), 5439-5447.
5. Aftab, U.; Tahira, A.; Mazzaro, R.; Abro, M. I.; Baloch, M. M.; Willander, M.; Nur, O.; Yu, C.; Ibupoto, Z. H., The Chemically Reduced CuO–Co_3O_4 Composite as A Highly Efficient Electrocatalyst for Oxygen Evolution Reaction in Alkaline Media. *Catal. Sci. Technol.* **2019,** *9* (22), 6274-6284.
6. Grewe, T.; Deng, X.; Tüysüz, H., Influence of Fe Doping on Structure and Water Oxidation Activity of Nanocast Co_3O_4. *Chem. Mater.* **2014,** *26* (10), 3162-3168.
7. Meng, J.; Cui, Z.; Yang, X.; Zhu, S.; Li, Z.; Qi, K.; Zheng, L.; Liang, Y., Cobalt-Iron (Oxides) Water Oxidation Catalysts: Tracking Catalyst Redox States and Reaction Dynamic Mechanism. *J. Catal.* **2018,** *365*, 227-237.

8. Waag, F.; Gökce, B.; Kalapu, C.; Bendt, G.; Salamon, S.; Landers, J.; Hagemann, U.; Heidelmann, M.; Schulz, S.; Wende, H.; Hartmann, N.; Behrens, M.; Barcikowski, S., Adjusting the Catalytic Properties of Cobalt Ferrite Nanoparticles by Pulsed Laser Fragmentation in Water with Defined Energy Dose. *Sci. Rep.* **2017,** *7* (1), 13161.
9. Jung, S.; McCrory, C. C. L.; Ferrer, I. M.; Peters, J. C.; Jaramillo, T. F., Benchmarking Nanoparticulate Metal Oxide Electrocatalysts for the Alkaline Water Oxidation Reaction. *J. Mater. Chem. A* **2016,** *4* (8), 3068-3076.
10. Bai, L.; Hsu, C.-S.; Alexander, D. T. L.; Chen, H. M.; Hu, X., A Cobalt–Iron Double-Atom Catalyst for the Oxygen Evolution Reaction. *J. Am. Chem. Soc.* **2019,** *141* (36), 14190-14199.
11. Gong, L.; Chng, X. Y. E.; Du, Y.; Xi, S.; Yeo, B. S., Enhanced Catalysis of the Electrochemical Oxygen Evolution Reaction by Iron(III) Ions Adsorbed on Amorphous Cobalt Oxide. *ACS Catal.* **2018,** *8* (2), 807-814.
12. Chang, C.-J.; Chu, Y.-C.; Yan, H.-Y.; Liao, Y.-F.; Chen, H. M., Revealing the Structural Transformation of Rutile RuO_2 via *in situ* X-ray Absorption Spectroscopy during the Oxygen Evolution Reaction. *Dalton Trans.* **2019,** *48* (21), 7122-7129.
13. Doyle, R. L.; Lyons, M. E. G., Kinetics and Mechanistic Aspects of the Oxygen Evolution Reaction at Hydrous Iron Oxide Films in Base. *J. Electrochem. Soc.* **2013,** *160* (2), H142-H154.
14. Bredar, A. R. C.; Chown, A. L.; Burton, A. R.; Farnum, B. H., Electrochemical Impedance Spectroscopy of Metal Oxide Electrodes for Energy Applications. *ACS Appl. Energy Mater.* **2020,** *3* (1), 66-98.
